# IL-33-primed NLRP3 inflammasome in basophils drives IL-1β production and initiates atopic dermatitis inflammation

**DOI:** 10.1038/s41420-025-02630-6

**Published:** 2025-07-27

**Authors:** Yoshitaka Gunji, Takayoshi Matsumura, Tadayoshi Karasawa, Takanori Komada, Chintogtokh Baatarjav, Satoko Komori, Hidetoshi Aizawa, Yoshiko Mizushina, Hidetoshi Tsuda, Kensuke Miyake, Takashi Maruyama, Tsukasa Ohmori, Hajime Karasuyama, Masafumi Takahashi

**Affiliations:** 1 Division of Inflammation Research, Center for Molecular Medicine, Jichi Medical University, Tochigi, Japan; 2 Division of Cardiovascular and Genetic Research, Center for Molecular Medicine, Jichi Medical University, Tochigi, Japan; 3 Division of Cardiovascular Medicine, Department of Medicine, Jichi Medical University, Tochigi, Japan; 4 Institute of Integrated Research, Institute of Science Tokyo, Tokyo, Japan; 5 Laboratory of Microbiology and Immunology, Graduate School of Pharmaceutical Sciences, Chiba University, Chiba, Japan; 6 Department of Biochemistry, Jichi Medical University, Tochigi, Japan

**Keywords:** Inflammasome, Inflammasome

## Abstract

Atopic dermatitis (AD) is a chronic inflammatory skin disorder caused by immune dysregulation that involves the release of various pro-inflammatory cytokines. Patients with AD frequently exhibit basophil infiltration in the affected skin. Although the role of the NLRP3 inflammasome in innate immune cells has been extensively studied, the contribution of the basophil inflammasome to the pathophysiology of AD remains to be elucidated. In this study, we demonstrated that IL-33 primes the NLRP3 inflammasome in basophils, leading to the production and release of mature IL-1β. Mechanistically, we showed that IL-33 stimulation induced pro-IL-1β and NLRP3 expression via the NF-κB and p38 MAPK pathways and that basophils released mature IL-1β through the canonical inflammasome activation pathway, which requires NLRP3, ASC, caspase-1, and gasdermin D (GSDMD). In an oxazolone (OXA)-induced AD mouse model, we found that basophils acted as key initiators of inflammation by producing IL-1β in the lesion, and that basophil depletion, genetic ablation of *Nlrp3* or *Il1b*, or basophil-specific genetic ablation of *Nlrp3* ameliorated ear swelling and neutrophil infiltration. Collectively, these findings establish basophils as a significant early source of NLRP3 inflammasome-driven IL-1β, contributing to the pathogenesis of AD. Targeting the IL-33/ST2L axis or NLRP3 inflammasome activation in basophils may offer a promising therapeutic strategy for managing AD.

## Introduction

Atopic dermatitis (AD) is a chronic inflammatory skin condition characterized by intense itching, erythema, and eczematous lesions, affecting 15–30% of children and 2–10% of adults in developed countries [1]. It is generally associated with a T helper 2 (Th2) immune response, leading to an imbalance in the immune system that promotes allergic inflammation. Recent studies have identified the distinct presence of neutrophils, particularly in the intrinsic type of AD, a non-IgE-mediated form of AD, or Asian AD [2–4]. Neutrophil-derived proteins, including myeloperoxidase, elastase, and lipocalin, contribute to pruritus progression during AD [5]. Several studies have also reported that neutrophil count and the neutrophil-to-lymphocyte ratio correlate with AD severity [6, 7]. However, the triggers for neutrophil infiltration and activation are not fully understood.

Interleukin-33 (IL-33), a member of the IL-1 superfamily, is a key cytokine that promotes the Th2 immune response. IL‑33 is constitutively expressed at high levels in the nuclei of various cell types, including endothelial cells, epithelial cells, and fibroblasts [8, 9], and is released as an alarm signal (alarmin) during cellular damage [10]. Unlike other IL-1 superfamily members, IL-1β and IL-18, a full-length IL-33 is biologically active [11] and binds to its specific receptor, ST2L, which forms a heterodimer with the IL-1 receptor accessory protein (IL-1RAcP) to mediate immune responses. Because ST2L is abundantly expressed on mast cells, group 2 innate lymphoid cells (ILC2s), regulatory T cells, and basophils [12], but is poorly expressed in neutrophils, it is important to clarify the cell-specific roles of IL-33 in allergic inflammation in AD. In a Phase 2a clinical trial of Etokimab, a monoclonal antibody targeting IL-33, significant reductions were observed in peripheral blood eosinophil and neutrophil infiltration in the skin of AD patients [13], suggesting that IL-33 contributes to both Th2-mediated eosinophilic infiltration and neutrophilic inflammation. However, the exact mechanism by which IL-33 facilitates neutrophil inflammation remains unclear.

Basophils are potential targets of IL-33. Basophils are the rarest granulocytes in the blood and account for <1% of the circulating leukocytes. Advances in techniques such as the use of basophil-deficient/reporter mice have revealed their significant roles in chronic allergic inflammation, autoimmune diseases, and protective immunity against parasitic infections [14–18]. AD patients frequently exhibit basophil infiltration in the affected skin lesions [19–21]. A recent study reported that skin-infiltrating basophils contribute to AD-like skin inflammation by producing IL-4 locally [22]. These findings led us to hypothesize that basophils trigger neutrophilic inflammation during the pathophysiology of AD.

The NLRP3 inflammasome is an intracellular molecular complex that plays a crucial role in mediating inflammation under various pathological conditions [23–26]. It is composed of a nucleotide-binding oligomerization domain-like receptor (NLR) family pyrin domain containing 3 (NLRP3), apoptosis-associated speck-like protein containing a caspase recruitment domain (ASC), and cysteine protease caspase-1. The assembly of the NLRP3 inflammasome results in the activation of caspase-1, which processes the pro-IL-1β and pro-IL-18 into their mature forms. Furthermore, activated caspase-1 processes gasdermin D (GSDMD), generating its N-terminal fragment (N-GSDMD), which forms pores in the cell membrane, facilitating the extracellular secretion of IL-1β and IL-18 and triggering pyroptosis, a form of inflammatory cell death [27]. A recent study revealed elevated levels of pro-inflammatory cytokines in the skin lesions of AD, including IL-1β and IL-18, suggesting a potential link between AD and the NLRP3 inflammasome [28]. However, no information is currently available regarding the role of NLRP3 inflammasome in AD.

In the present study, we investigated whether or not IL-33 could activate the NLRP3 inflammasome in basophils and contribute to neutrophilic inflammation and AD development. Our findings help clarify the role and mechanism of the NLRP3 inflammasome in the pathophysiology of AD and suggest that the NLRP3 inflammasome in basophils offers a promising therapeutic target for managing AD.

## Results

### IL-33 serves as a priming signal for NLRP3 inflammasome in basophils

**Fig. 1:**
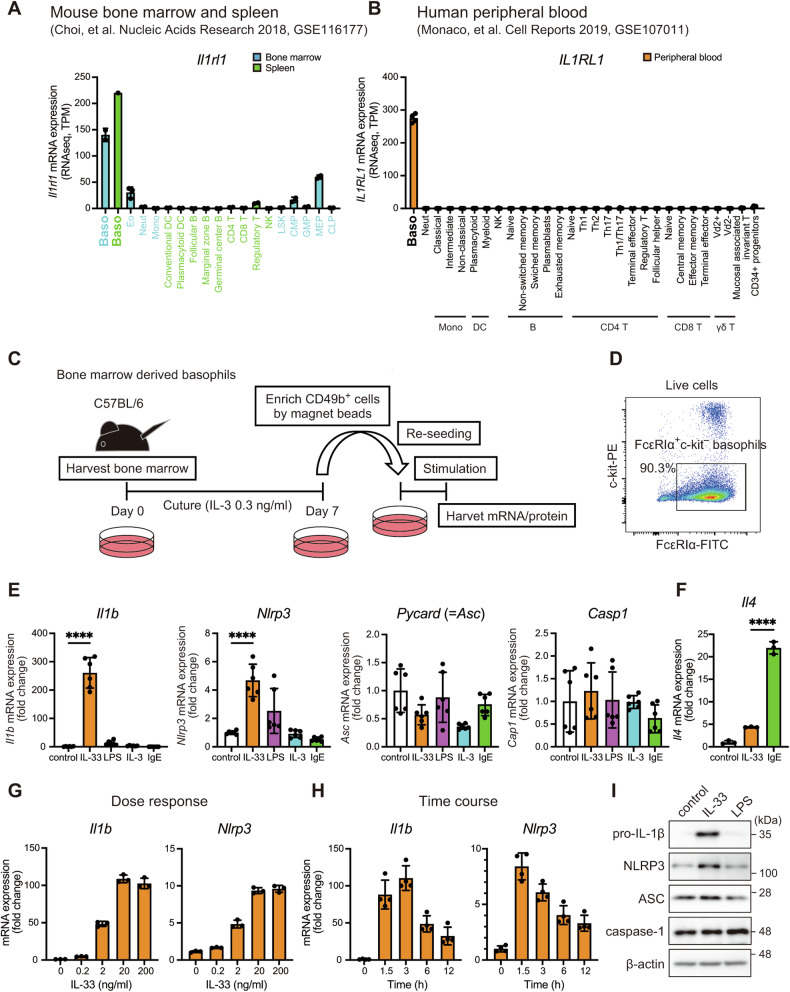
IL-33 serves as a priming signal for NLRP3 inflammasome in basophils. **A** An analysis of publicly available RNA-seq data (GSE116117) showing mRNA expression in mouse bone marrow and spleen (*n* = 1 for basophils of spleen, *n* = 3 for eosinophils, and *n* = 2 for others. *P* < 0.0001 by a one-way ANOVA). **B** An analysis of publicly available RNA-seq data showing mRNA expression in human peripheral blood (*n* = 2 for CD4^+^ terminal effector T cells, and *n* = 4 for others. *P* < 0.0001 by a one-way ANOVA). **C** Experimental design. Bone marrow cells were cultured in the presence of IL-3 (0.3 ng/ml) for 7 days, and CD49b^+^ cells were isolated. **D** The percentage of FcεRIα^+^ and c-kit^–^ (basophils) was analyzed by flow cytometry. **E** The expression of *Il1b*, *Nlrp3*, *Pycard*, and *Casp1* in BMBAs stimulated with IL-33 (20 ng/ml), LPS (300 ng/ml), IL-3 (20 ng/ml), or TNP-OVA (10 ng/ml) was assessed by real-time RT-PCR (*n* = 6). **F** The *Il4* expression in BMBAs stimulated with IL-33 or TNP-OVA was assessed by real-time RT-PCR (*n* = 3). **G**, **H** The expression of *Il1b* and *Nlrp3* in BMBAs stimulated with IL-33 was assessed by real-time RT-PCR (G: dose-response, *n* = 3; H: time course, *n* = 4). Data are expressed as dot plots with the mean ± SD. *P < 0.05, **P < 0.01, ***P < 0.001, ****P < 0.0001. **I** The protein levels of pro-IL-1β, NLRP3, ASC, caspase-1, and β-actin were assessed by immunoblotting.

To explore stimuli that act as a priming signal for the NLRP3 inflammasome in basophils, we first reanalyzed previously published RNA sequencing data obtained from mouse bone marrow, spleen, and human peripheral blood cells [29, 30]. Transcriptome data showed that basophils had the highest expression of *Il1rl1* (encoding ST2L) among mouse and human blood cells (Fig. 1A, B). Publicly available single-cell RNA-sequencing (scRNA-seq) data also showed that basophils and mast cells exhibited the highest *IL1RL1* expression among all human cell types profiled (Supplementary Fig. 1) [31]. The specific expression of *Il1rl1* in basophils is unique compared to the broader expression of other receptors, including *Il3ra* (the receptor for IL-3), *Fcer1a* (the receptor for IgE), and *Tlr4* (Supplementary Fig. 2A, B). Furthermore, basophils expressed all NLRP3 inflammasome components, *Nlrp3*, *Pycard* (encoding ASC), and *Casp1*, similar to neutrophils and monocytes (Supplementary Fig. 3A, B). These results prompted us to study whether and how IL-33 initiates the NLRP3 inflammasome and subsequently induces the secretion of mature IL-1β.

To analyze the NLRP3 inflammasome in basophils, we used bone marrow–derived basophils (BMBAs) (Fig. 1C, D). Among the stimuli examined, only IL-33 robustly increased the mRNA expression of *Il1b* and *Nlrp3* (Fig. 1E). LPS is a well-known priming activator of the NLRP3 inflammasome in macrophages and dendritic cells [32]; however LPS modestly upregulated *Nlrp3* and failed to stimulate *Il1b* expression. Under conditions where IgE cross-linking enhanced *Il4* significantly (Fig. 1F), neither high concentrations of IL-3 (20 ng/ml) nor IgE cross-linking induced the expression of *Il1b* and *Nlrp3* (Fig. 1E). *Pycard* and *Casp1* were not affected by any stimulus. IL-33-induced *Il1b* and *Nlrp3* expression in a dose- and time-dependent manner (Fig. 1G, H). We also assessed the protein levels of inflammasome-related molecules using immunoblotting. While ASC and caspase-1 were constitutively expressed in basophils, pro-IL-1β was undetectable in unstimulated cells (Fig. 1I). IL-33, but not LPS, potently induced the protein levels of pro-IL-1β and NLRP3, in line with the mRNA analyses. These findings indicated that IL-33 serves as an efficient priming signal for the NLRP3 inflammasome in basophils.

### IL-33 acts as a priming signal for NLRP3 inflammasome in lung basophils in vivo

**Fig. 2:**
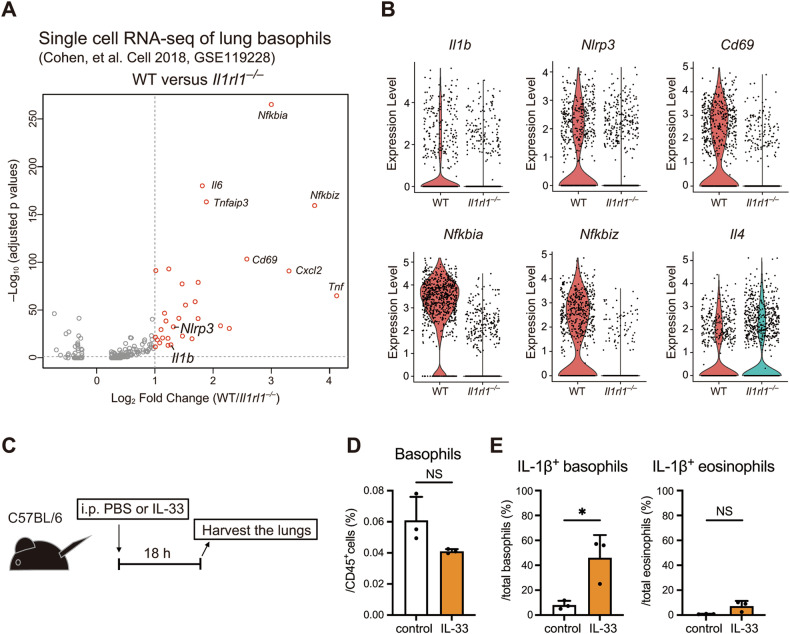
IL-33 acts as a priming signal for NLRP3 inflammasome in lung basophils in vivo. **A** Publicly available scRNA-seq data (GSE119228) were reanalyzed. Differential gene expression between 30 h postnatal lung basophils from WT versus *Il1rl1*^*–/–*^ mice are shown (x-axis). Adjusted *p* values are shown (y-axis). **B** Violin plots showing the expression of *Il1b*, *Nlrp3*, *Cd69*, *Nfkbia*, *Nfkbiz*, and *Il4* in WT and *Il1rl1*^*–/–*^ basophils are shown. **C** Experimental design. **D** The percentages of basophils among CD45⁺ cells in the control and IL-33-treated groups are shown (*n* = 3). **E** Intracellular IL-1β expression in basophils and eosinophils of the lungs was analyzed by flow cytometry. The ratios of IL-1β^+^ basophils and eosinophils are shown (*n* = 3). Data are expressed as dot plots with the mean ± SD. *P < 0.05.

We next studied whether or not IL-33 activates a priming signal for the NLRP3 inflammasome in vivo using scRNA-seq data of lung resident basophils obtained from postnatal wild-type (WT) and *Il1rl1*^*–/–*^ mice (Fig. 2A) [33]. *Il1b* and *Nlrp3* were upregulated in WT basophils compared to *Il1rl1*^*–/–*^ basophils, along with previously reported genes, including *Il6*, *Tnf*, *Cd69*, *Nfkbia* (encoding IκBα), and *Nfkbiz* (encoding IκBζ) (Fig. 2A, B). In contrast, while basophil-derived IL-4 has been reported to play a critical role in allergic lung injury and AD [22, 34], the *Il4* expression was comparable between WT and *Il1rl1*^*–/–*^ basophils, consistent with in vitro data showing that IL-33 alone did not strongly induce *Il4* expression (Fig. 1F). To confirm the direct effect of IL-33 on basophils in vivo, mice were administered IL-33 intraperitoneally, and lungs were collected 18 h later (Fig. 2C). Flow cytometry analysis revealed a significant increase in IL-1β expression in basophils in the IL-33-treated group compared with the control group (Fig. 2D, E). In contrast, eosinophils showed little to no increase in IL-1β expression. These findings revealed that IL-33 acts as a priming signal for the NLRP3 inflammasome in lung-resident basophils in vivo.

### IL-33 increases Il1b and Nlrp3 expression through NF-κB and p38 MAPK pathways

**Fig. 3:**
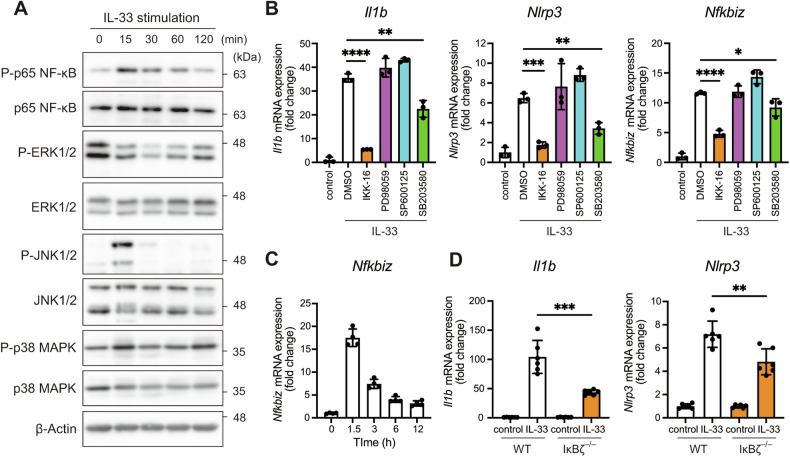
IL-33 elevates Il1b and Nlrp3 expression through NF-κB and p38 MAPK pathways. **A** BMBAs were stimulated with IL-33 for the indicated periods. The levels of P-p65 NF-κB, p65 NF-κB, P-ERK1/2, ERK1/2, P-JNK1/2, JNK1/2, P-p38 MAPK, p38 MAPK, and β-actin were assessed by immunoblotting. **B** BMBAs were pretreated with DMSO, IKK-16 (2 mM), PD98059 (10 mM), SP600125 (10 mM), and SB203580 (10 mM) and then stimulated with IL-33. The expression of *Il1b*, *Nlrp3*, *Nfkbia*, and *Nfkbiz* was assessed by real-time RT-PCR (*n* = 3). **C** The expression of *Nfkbiz* in BMBAs stimulated with IL-33 for the indicated periods was assessed by real-time RT-PCR (*n* = 4). **D** BMBAs from WT and *Mx1-Cre Nfkbiz* ^*flox/flox*^ mice were stimulated with IL-33. The expression of *Il1b* and *Nlrp3* was assessed by real-time RT-PCR. Data are expressed as dot plots with the mean ± SD. *P < 0.05, **P < 0.01, ***P < 0.001, ****P < 0.0001.

Previous studies have shown that IL-33-activated pathways differ between macrophages and mast cells [35–37]. Immunoblotting revealed that in basophils, IL-33 induced the phosphorylation of p65 NF-κB, JNK, and p38 MAPK, which peaked at 15 min after stimulation, whereas it suppressed the phosphorylation of ERK1/2 (Fig. 3A). IKK-16 (an inhibitor of the NF-κB pathway) nearly completely suppressed the expression of *Il1b* and *Nlrp3*, whereas SB203580 (an inhibitor of the p38 MAPK pathway) resulted in partial inhibition (Fig. 3B). In contrast, PD98059 (an inhibitor of the ERK1/2 pathway) and SP600125 (an inhibitor of the JNK pathway) did not affect the expression of *Il1b* and *Nlrp3*. Thus, the expression of pro-IL-1β and NLRP3 induced by IL-33 is mediated through the NF-κB and p38 MAPK pathways.

IκBζ transcriptionally regulates various cytokine genes, particularly IL-6, in macrophages [38]. Regarding IL-33 signaling, IκBζ has also been shown to regulate cytokine genes such as IL-6 and IL-13 in mast cells [36]. In basophils, we found that IL-33 increased the *Nfkbiz* expression in BMBAs, which was significantly inhibited by IKK-16 (Fig. 3B, C). We further examined the role of IκBζ in *Mx1-Cre Nfkbiz*^*fl/fl*^ mice, which showed hematopoietic cell-specific deletion of *Nfkbiz* after pIpC injection. BMBAs derived from *Mx1-Cre Nfkbiz*^*fl/fl*^ mice exhibited a significantly reduced expression of *Il1b* and *Nlrp3* after IL-33 stimulation, compared with WT BMBAs (Fig. 3D). These results suggest that NF-κB regulates the IκBζ expression, which also contributes to IL-33-induced priming of the NLRP3 inflammasome in basophils.

### Basophils produce mature IL-1β through NLRP3 inflammasome

**Fig. 4:**
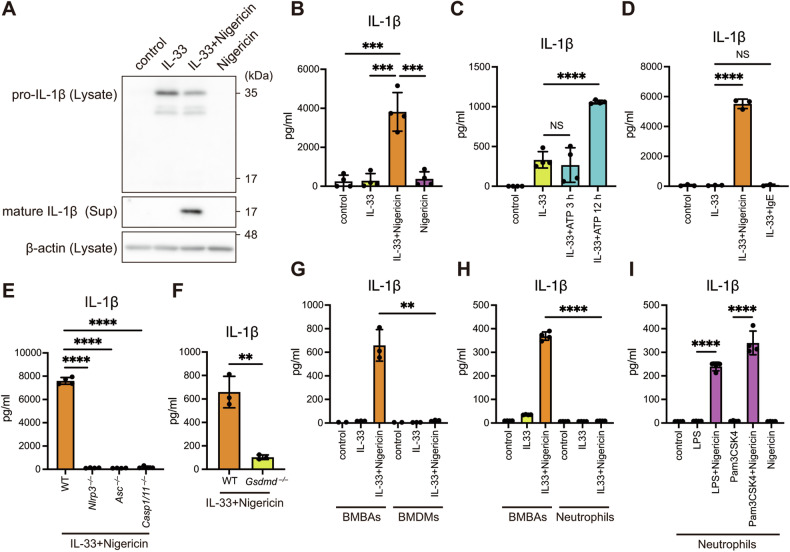
Basophils produce mature IL-1β through NLRP3 inflammasome. **A**, **B** BMBAs primed with IL-33 for 6 h were stimulated with nigericin (10 mM) for 1 h. Cell lysates and supernatants were prepared. The protein levels of pro-IL-1β, mature IL-1β, and β-actin were assessed by immunoblotting (**A**). **B** IL-1β levels in the supernatants were assessed by an ELISA (*n* = 4). **C** BMBAs primed with IL-33 for 18 h were stimulated with ATP (5 mM) for 3 or 12 h. IL-1β levels in the supernatants were assessed (*n* = 4). **D** BMBAs primed with IL-33 for 6 h were stimulated with nigericin for 1 h or TNP-OVA for 3 h. IL-1β levels in the supernatants were assessed (*n* = 3). **E** IL-1β levels in the supernatants of WT, *Nlrp3*^*–/–*^, *Asc*^*–/–*^, and *Casp1/11*^*–/–*^ BMBAs were assessed (*n* = 4). **F** IL-1β levels in the supernatants of WT and *Gsdmd*^*–/–*^ BMBAs were assessed (*n* = 4). **G** IL-1β levels in the supernatants of BMBAs and BMDMs were assessed (*n* = 3). **H** IL-1β levels in the supernatants of BMBAs and neutrophils were assessed (*n* = 4). **I** Neutrophils primed with LPS or Pam3CSK4 for 6 h were stimulated with nigericin for 1 h. IL-1β levels in the supernatants of neutrophils were assessed (*n* = 4). Data are expressed as dot plots with the mean ± SD. *P < 0.05, **P < 0.01, ***P < 0.001, ****P < 0.0001.

NLRP3 inflammasome-driven mature IL-1β secretion requires two signals: the priming signal to enhance the transcription of *Il1b* and the secondary signal to process pro-IL-1β to its mature form [39]. Because IL-33 alone did not induce the processing of pro-IL-1β in basophils (Fig. 4A), we tested the effect of known caspase-1 activating stimuli. Nigericin is known to activate the NLRP3 inflammasome through potassium (K^+^) efflux in macrophages and dendritic cells [32]. In basophils, nigericin stimulation for 1 h after IL-33 priming led to pro-IL-1β processing and secretion of mature IL-1β, as determined by immunoblotting (Fig. 4A) and an enzyme-linked immunosorbent assay (ELISA) (Fig. 4B). ATP is also known as an activator of the NLRP3 inflammasome [40], and its stimulation after LPS priming induces mature IL-1β secretion within 30 min in macrophages and dendritic cells [32]. In contrast to the findings for nigericin stimulation. ATP after IL-33 priming did not induce mature IL-1β secretion within 3 h, but long-term ATP stimulation for 12 h induced its secretion in BMBAs (Fig. 4C). This observation indicates that although ATP can act as a secondary signal for the NLRP3 inflammasome in both macrophages and basophils, the underlying mechanisms likely differ between the two cell types. Given previous reports of IL-1β secretion in mast cells in response to IgE cross-linking [41], we tested IgE cross-linking stimulation; however, no IL-1β was detected (Fig. 4D).

In canonical NLRP3 inflammasome activation, ASC recruits pro-caspase-1, and then pro-caspase-1 self-hydrolyzes to active caspase-1, which in turn converts pro-IL-1β to mature IL-1β [24]. In addition, IL-1β secretion independent of caspase-1 has also been reported in macrophages and dendritic cells [42, 43]. Therefore, we investigated whether or not each NLRP3 inflammasome component was involved in IL-1β production using *Nlrp3*^*–/–*^, *Asc*^*–/–*^, and *Casp1/11*^*–/–*^ mice. The secretion of mature IL-1β was completely abolished in BMBAs from *Nlrp3*^*–/–*^, *Asc*^*–/–*^, and *Casp1/11*^*–/–*^ mice, indicating that IL-33/nigericin-induced mature IL-1β production in basophils requires the canonical NLRP3 inflammasome (Fig. 4E). We also observed that mature IL-1β secretion was significantly suppressed in BMBAs from *Gsdmd*^*–/–*^ mice, suggesting that IL-1β was secreted mainly through GSDMD-forming pores (Fig. 4F).

Considering the high expression of *Il1rl1* on basophils among blood cells (Fig. 1A, B), we hypothesized that basophils might produce IL-1β more than other cell types under conditions rich in IL-33. We compared the reactivities of BMBAs and bone marrow-derived macrophages (BMDMs) with the same number of cells. Upon stimulation of IL-33-primed BMBAs and BMDMs with nigericin, IL-1β production was detected only in BMBAs and was almost negligible in BMDMs (Fig. 4G). In neutrophils, while TLR2/4 stimulation by Pam3CSK4 or LPS, followed by nigericin, induced robust IL-1β secretion, IL-33-primed neutrophils failed to produce IL-1β (Fig. 4H, I), suggesting that TLR signaling is required for inflammasome priming in neutrophils. These findings suggest that basophils play a critical role as sources of IL-1β in IL-33-rich environments.

### Basophils are recruited and enhance the Il1b expression in an AD mouse model

**Fig. 5:**
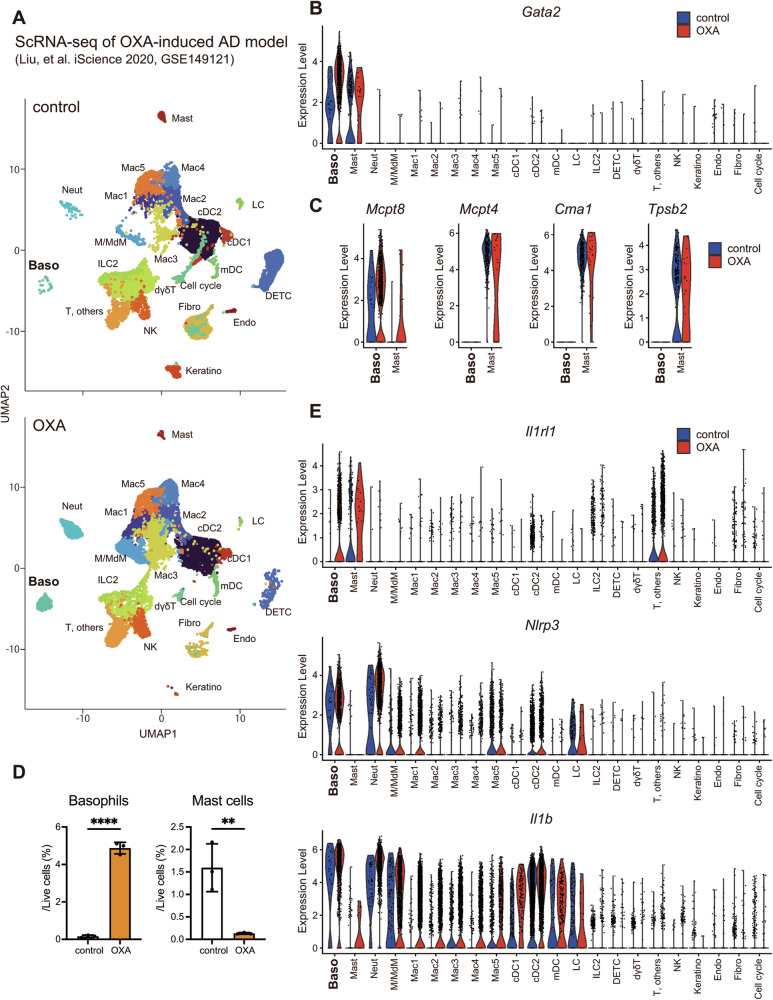
Basophils are recruited and enhance Il1b expression in an AD mouse model. **A** Publicly available scRNA-seq data (GSE149121) were reanalyzed. UMAP plot of the control (ethanol-treated) and OXA-treated skin samples are shown. Colors represent different Seurat clusters. **B** Violin plots showing the expression of *Gata2* in control and OXA-treated skin samples are shown. **C** Violin plots showing the expression of *Mcpt8*, *Mcpt4, Cma1*, and *Tpsb2* in control and OXA-treated skin samples are shown. **D** The ratios of basophils and mast cells in the ear skin samples are shown (*n* = 3). Data are expressed as dot plots with the mean ± SD. *P < 0.05, **P < 0.01, ***P < 0.001, ****P < 0.0001. **E** Violin plots showing the expression of *Il1rl1*, *Nlrp3*, and *Il1b* in control and OXA-treated skin samples are shown.

Basophils are absent in healthy human skin. However, basophil recruitment is a common feature in the lesional skin of patients with AD [19–21]. To elucidate the role of IL-1β produced by basophils under pathological conditions, we reanalyzed publicly available scRNA-seq data from an OXA-induced AD mouse model (Fig. 5A and Supplementary Fig. 4) [44]. OXA is a well-known hapten that has been widely used in AD models, as it markedly upregulates IL-33 and induces skin inflammation characterized by a Th2 immune response [22, 45–49]. Notably, we successfully distinguished two clusters with high *Gata2* and *Fcer1a* expression: the basophil cluster with an increased expression of *Mcpt8*, and the mast cell cluster with the exclusive expression of *Cma1*, *Mcpt4*, and *Tpsb2* (Fig. 5B, C and Supplementary Fig. 5). This discrimination allowed us to confirm that basophils were recruited into the skin lesions in this AD model (Fig. 5D). Mast cells were not increased, and eosinophils were not detected. Furthermore, while basophils, mast cells, and a subset of T cells showed the induction of *Il1rl1* upon OXA stimulation, only basophils showed the concomitant expression of *Nlrp3* among these cells. While basophils had high basal expression and OXA-induced upregulation of *Il1b* but not *Il18*, *Il1b* expression was much lower in mast cells and T cells, suggesting that these cells were unlikely to secrete sufficient amounts of IL-1β in response to IL-33 (Fig. 5E and Supplementary Fig. 5). Taken together, these transcriptome data imply that IL-1β from basophils, rather than mast cells, plays a crucial role in the initiation of IL-33-driven inflammatory conditions.

### Depletion of basophils is sufficient to ameliorate the inflammation in the AD model

**Fig. 6:**
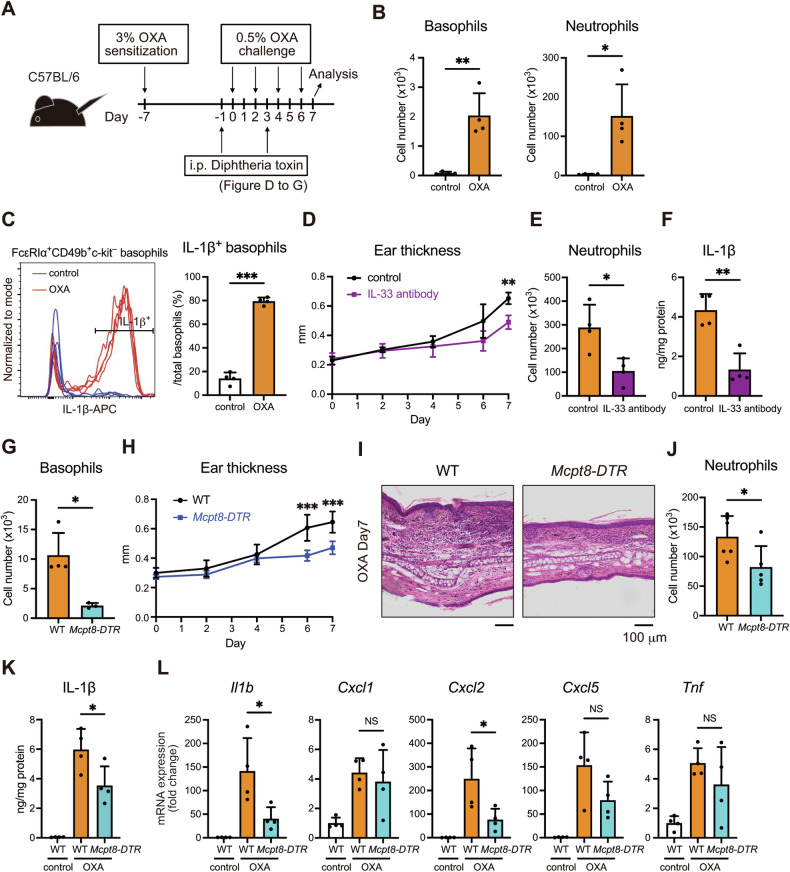
Deletion of basophils ameliorates the inflammation in the AD model. **A** Experimental design. **B** The number of basophils and neutrophils of the ear skin samples on day 7 is shown (*n* = 4). **C** Intracellular IL-1β expression in basophils of the ear skin samples was analyzed by flow cytometry. The ratio of IL-1β^+^ basophils is shown (*n* = 4). **D** On day 2, mice were intraperitoneally injected with either an isotype control or anti-IL-33 neutralizing antibody. The time course of ear thickening is shown (*n* = 4). **E**, **F** The number of neutrophils and IL-1β levels in the ear skin samples on day 7 is shown (*n* = 4). **G** The number of basophils in the ear skin samples on day 7 is shown (*n* = 4 for WT, *n* = 3 for *Mcpt8-DTR* mice). **H** The time course of ear thickening in WT and *Mcpt8-DTR* mice is shown (*n* = 6 for WT, *n* = 7 for *Mcpt8-DTR* mice). **I** Representative HE staining of ear samples from WT and *Mcpt8-DTR* mice is shown. **J** The number of neutrophils in the ear skin samples on day 7 is shown (*n* = 6 for WT, *n* = 5 for *Mcpt8-DTR* mice). **K** IL-1β levels in the ear skin samples on day 7 were assessed (*n* = 4). **L** The expression of *Il1b*, *Cxcl1*, *Cxcl2*, *Cxcl5*, and *Tnf* in the ear skin samples on day 7 was assessed by real-time RT-PCR (*n* = 4). Data are expressed as dot plots with the mean ± SD. *P < 0.05, **P < 0.01, ***P < 0.001.

To investigate the role of basophil inflammasome in the development of AD, we also conducted an OXA-induced AD model experiment, and revealed a marked infiltration of basophils and neutrophils in the skin lesion in WT mice, consistent with the scRNA-seq data (Fig. 6A, B). Intriguingly, most infiltrating basophils expressed IL-1β at the protein level (Fig. 6C). To examine the importance of IL-33 in this model, we administered an IL-33 neutralizing antibody. IL-33 blockade significantly reduced ear swelling, neutrophil infiltration, and IL-1β expression in the lesional skin (Fig. 6D–F), suggesting that IL-33 is a key driver of basophil activation in this context. Next, we utilized *Mcpt8-DTR* mice, in which diphtheria toxin selectively ablated basophils (Fig. 6G). Consistent with our previous report [22], *Mcpt8-DTR* mice exhibited reduced ear thickening and attenuated neutrophil infiltration compared with WT mice (Fig. 6H–J). Notably, IL-1β protein induction by OXA in the ear was almost halved in *Mcpt8-DTR* mice (Fig. 6K). Furthermore, the expression of *Cxcl2*, a neutrophil-recruiting chemokine induced by IL-1β [50, 51], was significantly reduced in the skin lesions in *Mcpt8-DTR* mice (Fig. 6L), suggesting IL-1β promotes neutrophil infiltration partly via CXCL2 in this model. These results indicate that depletion of basophils is sufficient to suppress neutrophil recruitment and ameliorate local IL-1β production despite their far smaller population than neutrophils, indicating their critical role as initiators of AD pathogenesis.

### NLRP3 inflammasome in basophils contributes to neutrophilic inflammation in the AD model

**Fig. 7:**
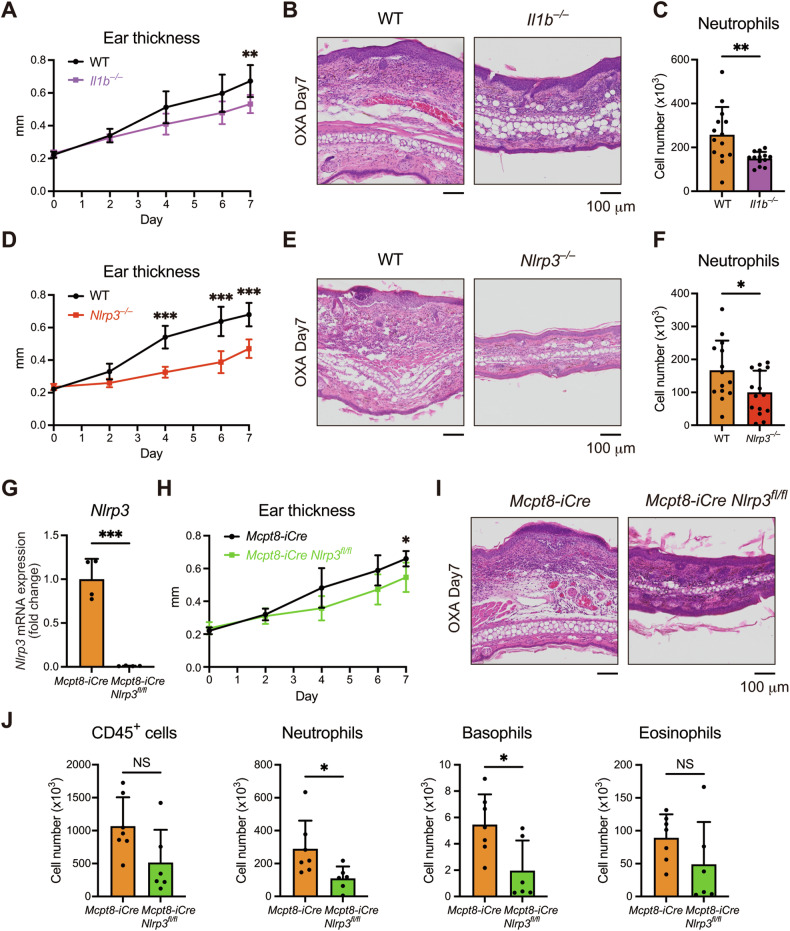
NLRP3 inflammasome in basophils contributes to neutrophilic inflammation in the AD model. **A** The time course of ear thickening in WT and *Il1b*^*–/–*^ mice is shown (*n* = 9 for each). **B** Representative HE staining of ear samples from WT and *Il1b*^*–/–*^ mice is shown. **C** The number of neutrophils in the ear skin samples on day 7 is shown (*n* = 14 for WT, 13 for *Il1b*^*–/–*^ mice). **D** The time course of ear thickening in WT and *Nlrp3*^*–/–*^ mice is shown (*n* = 9 for WT, *n* = 10 for *Nlrp3*^*–/–*^ mice). **E** Representative HE staining of ear samples from WT and *Nlrp3*^*–/–*^ mice is shown. **F** The number of neutrophils in the ear skin samples on day 7 is shown (*n* = 14 for WT, *n* = 15 for *Nlrp3*^*–/–*^ mice). **G** The expression of *Nlrp3* in BMBAs from *Mcpt8-iCre* mice and *Mcpt8-iCre Nlrp3*^*fl/fl*^ mice was assessed by real-time RT-PCR (*n* = 4). **H** The time course of ear thickening in *Mcpt8-iCre* and *Mcpt8-iCre Nlrp3*^*fl/fl*^ mice is shown (*n* = 5). **I** Representative HE staining of ear samples from *Mcpt8-iCre* and *Mcpt8-iCre Nlrp3*^*fl/fl*^ mice is shown. **J** The number of CD45^+^ cells, neutrophils, basophils, and eosinophils in the ear skin samples on day 7 is shown (*n* = 7 for *Mcpt8-iCre*, *n* = 6 for *Mcpt8-iCre Nlrp3*^*fl/fl*^ mice). Data are expressed as dot plots with the mean ± SD. *P < 0.05, **P < 0.01, ***P < 0.001.

To elucidate the role of the NLRP3 inflammasome in the development of AD, we also examined *Il1b*^*–/–*^ and *Nlrp3*^*–/–*^ mice. The absence of IL-1β reduced ear thickening and neutrophil infiltration in the skin lesions after OXA application (Fig. 7A–C). Consistently, NLRP3 deficiency led to a significant reduction in ear thickening and neutrophil infiltration, indicating the critical role of the NLRP3 inflammasome in promoting inflammation in this model (Fig. 7D–F).

Finally, to directly assess the function of the NLRP3 inflammasome in basophils, we generated basophil-specific NLRP3-deficient mice by crossing *Mcpt8-iCre* mice [52] and *Nlrp3*^*fl/fl*^ mice [53]. We confirmed the complete suppression of *Nlrp3* expression in BMBAs from *Mcpt8-iCre Nlrp3*^*fl/fl*^ mice (Fig. 7G). Notably, consistent with the findings from *Il1b*^*–/–*^ and *Nlrp3*^*–/–*^ mice, *Mcpt8-iCre Nlrp3*^*fl/fl*^ mice exhibited a significant reduction in ear thickening (Fig. 7H-I). Furthermore, NLRP3 deficiency only in basophils led to a substantial decrease in inflammatory cell infiltration, including neutrophils and basophils (Fig. 7J). Taken together, these results suggest the pivotal role of basophil-derived IL-1β, regulated by the NLRP3 inflammasome, in orchestrating neutrophilic inflammation and initiating AD pathogenesis.

## Discussion

In the present study, we revealed a novel role for basophils in AD pathogenesis by demonstrating that IL-33 primes the NLRP3 inflammasome and facilitates the production of mature IL-1β. Traditionally, basophils have been recognized as key mediators of type 2 immune responses in allergic diseases through the release of histamine and other mediators [17]. However, emerging evidence suggests that basophils participate in immune regulation [54, 55]. Our results align with those of previous studies that have identified IL-33 as a critical factor in chronic allergic inflammation, including asthma and AD, where it activates innate immune cells to amplify inflammation [56, 57]. Although NLRP3 inflammasome activation has been studied extensively in macrophages and dendritic cells, its role in basophils has been poorly explored thus far [32]. This study addresses this gap by highlighting basophils as an important innate immune cell type contributing to inflammasome-driven allergic inflammation.

Our findings significantly expand the established roles of basophils by revealing that they are directly primed by IL-33 alone for inflammasome activation. To our knowledge, IL-33 has not been reported to be a priming signal for the NLRP3 inflammasome in macrophages or other cell types. Furthermore, although a previous study has shown that basophils can be primed with LPS and subsequently activated by Alum [58], our results suggest that IL-33 is a more effective priming signal than LPS in basophils. These novel findings highlight the uniqueness of IL-33-driven NLRP3 inflammasome activation in basophils and explain why they are critical regulators of inflammation in IL-33-rich environments.

Mechanistically, we found that IL-33 primed basophils via the NF-κB and p38 MAPK pathways, leading to the expression of pro-IL-1β and NLRP3. Notably, while IκBζ, an NF-κB-induced transcriptional regulator, has been documented in macrophages and mast cells, its function in basophils has not been well investigated. We demonstrated its role in priming the NLRP3 inflammasome in basophils using mice deficient in IκBζ. Furthermore, we confirmed that basophils released mature IL-1β through the classical inflammasome activation pathway, which requires NLRP3, ASC, caspase-1, and GSDMD, by examining knockout mice of each gene.

Although increased IL-1β expression has been reported in the skin of patients with AD, the role of the NLRP3 inflammasome and IL-1β in AD pathogenesis is unclear. Our findings imply that basophil-derived IL-1β plays a critical role in recruiting neutrophils to lesions and driving AD pathology. In the OXA-induced AD model, we observed a significant reduction in ear thickness and neutrophil infiltration following basophil depletion, or genetic ablation of *Nlrp3* and *Il1b*, confirming their contribution to neutrophilic inflammation. Furthermore, ear thickening and neutrophil infiltration were attenuated in basophil-specific NLRP3-deficient mice. These results are consistent with those of previous studies demonstrating the importance of IL-33 in AD and introducing a unique mechanism of basophils as initiators and neutrophils as executors in which basophils amplify neutrophil-driven inflammatory responses beyond their conventional Th2 cytokine-mediated allergic functions.

However, several limitations associated with the present study warrant mention. First, the experiments were performed using a murine AD model, and it remains to be determined whether or not similar mechanisms are involved in human basophils in the skin lesions of patients with AD. In addition, although the NLRP3 inflammasome activation in vitro was induced by nigericin and ATP as secondary signals in our study, the specific factors that serve as secondary signals in AD patients remain unidentified. Environmental factors, such as allergens, pollutants, or microbial exposure, may also influence basophil activation and contribute to AD, which could be a focus of future research.

In summary, this study identified a previously unrecognized role for basophils in the pathogenesis of AD-like skin lesions by demonstrating their ability to produce IL-1β through NLRP3 inflammasome activation in response to IL-33. These findings expand our understanding of basophil biology and offer new insights into innate immune mechanisms underlying AD. Targeting the IL-33/ST2L axis and the NLRP3 inflammasome in basophils may present promising therapeutic strategies for managing inflammation in AD. Although anti-IL-33 therapy showed limited efficacy in patients with chronic AD [59], our findings suggest IL-33 acts as an early initiator of inflammation. Thus, IL-33 inhibition may be more effective in early or milder stages of the disease. Future research is essential to validate these findings in human patients and explore additional pathways through which basophils contribute to immune regulation in allergic diseases.

## Materials and methods

### Animals and OXA-induced AD model

C57BL/6 J WT mice were purchased from SLC Inc. (Shizuoka, Japan). *Nlrp3*^*–/–*^, *Asc*^*–/–*^, *Casp1/11*^*–/–*^, *Il1b*^*–/–*^, and *Gsdmd*^*–/–*^ mice were kindly provided by Dr. V. M. Dixit (Genentech, San Francisco, CA, USA), S. Taniguchi (Shinshu University, Nagano, Japan), H. Tsutsui (Hyogo Medical College, Hyogo, Japan), Y. Iwakura (Tokyo University of Science, Chiba, Japan), and Genentech, respectively [60–64]. *Mcpt8-DTR* mice, *Mcpt8-iCre mice*, *Nlrp3*^*fl/fl*^ mice, and *Mx1-Cre Nfkbiz*^*fl/fl*^ mice were generated as previously described [36, 52, 53, 65]. Male mice, 8–10 weeks old, were used in this study. The mice were housed (RAIR HD-ventilated micro-isolator animal housing systems; Laboratory Products, Seaford, DE, USA) in an environment maintained at 23 ± 2˚C with ad libitum access to food and water under a 12-h light/dark cycle, with lights on from 8:00 to 20:00. Mice were randomly assigned to experimental groups. No blinding was performed during animal experiments or primary cell isolation.

To assess IL-1β expression in basophils in vivo, PBS or IL-33 was administered intraperitoneally, and the lungs were collected 18 h later. For an OXA-induced AD mouse model, mice were epicutaneously sensitized with 100 ml of 3% OXA (Sigma-Aldrich, St. Louis, MO, USA) on their abdomen 7 days before the first challenge and challenged every other day with 10 ml of 0.5% OXA and vehicle alone on their ears. Ear thickness was measured at the indicated time points. For basophil depletion, *Mcpt8-DTR* mice were intraperitoneally injected with 500 ng of diphtheria toxin (Bioacademia, Athens, Greece) 1 day before and 3 days after the first OXA challenge. For IL-33 neutralization, mice were intraperitoneally injected on day 2 with either an anti-mouse IL-33 antibody (100 μg; Medical & Biological Laboratories, Nagoya, Japan, M187-3) or an IgG isotype control antibody (100 μg; Vector Laboratories, Newark, CA, USA, I-2000-1).

### Hematoxylin and eosin (HE) staining

The ear was fixed in 10% formalin and embedded in paraffin. Tissue sections (4 mm thick) were stained with HE. Images of the stained sections were digitized and analyzed using a VS120 microscope (Olympus, Tokyo, Japan).

### Generation and stimulation of BMBAs and BMDMs

For BMBA culture, bone marrow cells were isolated from femurs, tibias, and hip bones, and cultured in RPMI-1640 (Wako, Osaka, Japan) supplemented with 10% fetal bovine serum (FBS), Antibiotic Antimycotic Solution (Sigma), and murine recombinant IL-3 (mrIL-3, 0.3 ng/ml; PeproTech, Cranbury, NJ, USA) for 7 days. CD49b^+^ cells were enriched using a biotinylated anti-CD49b antibody (DX5) (BioLegend, San Diego, CA, USA) and Mojosort streptavidin nanoparticles (BioLegend). For BMDM culture, bone marrow cells were cultured in RPMI-1640 (Wako) supplemented with 10% FBS, Antibiotic Antimycotic Solution (Sigma), and 15% conditioned medium from L929 cells (ATCC, Rockville, MD, USA) for 7 days. Neutrophils were isolated from bone marrow cells by density gradient centrifugation using 62% Percoll (Cytiva, Marlborough, MA, USA). After centrifugation, the neutrophil-rich fraction was collected from the bottom layer. Subsequently, BMBAs, BMDMs and neutrophils were incubated at 37˚C with IL-33 (20 ng/ml; BioLegend), mrIL-3 (20 ng/ml; PeproTech), lipopolysaccharide (LPS; 300 ng/ml; Sigma), Pam3CSK4 (300 ng/ml; InvivoGen, San Diego, CA, USA), or vehicles (PBS) for 3 h for mRNA analyses or for 6 h for protein analyses. To assess the involvement of NF-κB and MAPK pathways, BMBAs were pretreated with DMSO, IKK-16 (2 mM; Cayman Chemical Company, Ann Arbor, MI, USA), PD98059 (10 mM; EMD Millipore, Darmstadt, Germany), SP600125 (10 mM; EMD Millipore), and SB203580 (10 mM; EMD Millipore) for 1 h before stimulation. For immunoblotting and an ELISA, BMBAs, BMDMs or neutrophils were further stimulated with nigericin (10 mM; InvivoGen) for 1 h, or ATP (5 mM; Sigma) for 3 or 12 h. For IgE-antigen stimulation, BMBAs were sensitized overnight with TNP-specific IgE antibody (1 mg/ml; BioLegend) and were stimulated with TNP-conjugated ovalbumin (TNP-OVA; 10 ng/ml; Biosearch Technologies, Petaluma, CA, USA) for 3 h.

### Flow cytometric analyses

The lungs and the ear were incubated in complete RPMI-1640 supplemented with 10% FBS, and Antibiotic Antimycotic Solution containing Collagenase S-1 (2 mg/ml; Nitta Geratin Inc., Osaka, Japan) for 3 h. The cells were dissociated into a single-cell suspension by filtering through a 70-μm nylon cell strainer. The cells were Fc-blocked and stained with anti-mouse primary antibody for 30 min. The following antibodies were used: CD11b (M1/70) (BD Biosciences, San Jose, CA, USA, 557396/562605/562950), CD45 (30-F11) (BD, 553080/562891), CD49b (DX5) (BioLegend, 108921), CD117 (c-kit) (2B8) (BioLegend, 105826), FcεRIα (MAR-1) (BioLegend, 134308; eBioscience, San Diego, CA, USA, 11-5898-82), Ly6G (1A8) (BD, 560599; BioLegend, 127613), and SiglecF (S17007L, E50-2440) (BioLegend, 155506; BD Biosciences, 562661). For intracellular staining, cells were stained using Cytofix/Cytoperm Fixation/Permeabilization Solution (BD Biosciences). The cells were analyzed using FACS Verse or Lyric Cytometers (BD Biosciences). Each cell lineage was defined as follows: basophils (CD45^+^ FcεRIα^+^c-kit^–^CD49b^+^), neutrophils (CD45^+^Ly6G^high^), and eosinophils (CD45^+^Ly6G^–^SiglecF^+^). Data analyses were performed using the FlowJo analysis software program (FlowJo, LLC, Ashland, OR, USA).

### Real-time reverse transcription-polymerase chain reaction (RT-PCR)

Total RNA was prepared using the FastGene RNA Premium Kit (Nippon Gene Co., Tokyo, Japan) according to the manufacturer’s instructions. Total RNA was reverse-transcribed using SuperScript VILO Master Mix (Thermo Fisher Scientific, Waltham, MA, USA). Real-time RT-PCR was performed using TB Green Premix Ex Taq II (Tli RNaseH Plus) (Takara Bio Inc., Shiga, Japan) and Takara TP960 PCR Thermal Cycler Dice Real Time System II (Takara). The following primers were used: *Actb* forward: “CACAGCTTCTTTGCAGCTCCTT,” *Actb* reverse: “AGCGCAGCGATATCGTCAT,” *Il1b* forward: “TGAAGTTGACGGACCCCAAA,” *Il1b* reverse: “TGATGTGCTGCTGTGAGATT,” *Nlrp3* forward: “CGAGACCTCTGGGAAAAAGCT,” *Nlrp3* reverse: “GCATACCATAGAGGAATGTGATGTACA,” *Pycard* forward: “GCTGAGCAGCTGCAAACGAC,” *Pycard* reverse: “ACTTCTGTGACCCTGGCAATGAG,” *Casp1* forward: “GATGGCACATTTCCAGGACTGA,” *Casp1* reverse: “TGTTGCAGATAATGAGGGCAAGAC,” *Il4* forward: “GGTCTCAACCCCCAGCTAGT,” *Il4* reverse: “GCCGATGATCTCTCTCAAGTGAT,” *Nfkbia* forward: “CGAGACTTTCGAGGAAATACCC,” *Nfkbia* reverse: “GTCTGCGTCAAGACTGCTCA,” *Nfkbiz* forward: “GCTCCGACTCCTCCGATTTC,” and *Nfkbiz* reverse: “GAGTTCTTCACGCGAACACC,” *Cxcl1* forward: “GCTGGGATTCACCTCAAGAA,” *Cxcl1* reverse: “TCTCCGTTACTTGGGGACAC,” *Cxcl2* forward: “CGCTGTCAATGCCTGAAG,” *Cxcl2* reverse: “GGCGTCACACTCAAGCTCT,” *Cxcl5* forward: “TGCCCTACGGTGGAAGTCATA,” *Cxcl5* reverse: “TGCATTCCGCTTAGCTTTCTTT,” *Tnf* forward: “CCCCAAAGGGATGAGAAGTTC,” *Tnf* reverse: “GCTTGTCACTCGAATTTTGAGAA,” The expression of each target gene was normalized to *Actb* by using the ΔΔCT comparison method.

### Immunoblotting

Protein samples were denatured by boiling at 95 °C for 5 min under reducing conditions and then subjected to sodium dodecyl sulfate-polyacrylamide gel electrophoresis (SDS-PAGE). Protein bands were transferred onto polyvinylidene fluoride membranes (PVDF). The membranes were blocked for 1 h at room temperature with Blocking One or Blocking One-P (Nacalai tesque, Kyoto, Japan) and then reacted with the primary antibodies for 1 h, followed by incubation for 1 h with the secondary antibodies conjugated with horse-radish peroxidase (HRP). The following antibodies were used: IL-1β (R&D Systems, Minneapolis, MN, USA), NLRP3 (AdipoGen Life Sciences, San Diego, CA, USA), ASC (Cell Signaling Technology, Danvers, MA, USA), caspase-1 (AdipoGen), phosphorylated (P)-p65 NF-κB (Ser 536, Cell Signaling), p65 NF-κB (Santa Cruz Biotechnology, Dallas, TX, USA), P-ERK1/2 (Thr202/Tyr204; Cell Signaling), ERK1/2 (Cell Signaling), P-JNK (Thr183/Tyr185, Cell signaling), JNK (Santa Cruz), P-p38 MAPK (Thr180/Tyr182; Cell Signaling), p38 MAPK (Cell Signaling), mature-IL-1β (Asp117; Cell Signaling) and β-actin (Sigma). Western blot Quant or Ultra-Sensitive HRP substrate (Takara Bio Inc.) was used to detect the bands using an Amersham imager 680 (Cytiva, Marlborough, MA, USA). β-actin was used as an internal control for protein loading. Uncropped immunoblot images are provided in the Supplementary information.

### ELISA

The IL-1β levels in the culture supernatants or ear lysates were measured using a mouse ELISA kit (R&D Systems).

### ScRNA-seq data analysis

Previously published scRNA-seq data (GSE119228 and GSE149121) were downloaded and analyzed as previously described [66]. In brief, the Seurat package [67] in R was used. For skin scRNA-seq, clusters were detected using FindClusters and annotated based on the feature genes. Differentially expressed genes (DEGs) were identified using FindAllMarkers. Publicly available scRNA-seq data from the Tabula Sapiens project [31] were visualized using the CZ CELLxGENE Discover’s website [68].

### Statistical analyses

Data are presented as the means ± standard deviation (SD) from at least three independent experiments. All data were obtained from independent samples, and no data were excluded from the analysis. Sample sizes were based on standard protocols in the field. A statistical analysis was performed using a two-tailed Student’s t-test and analysis of variance (ANOVA) with the Tukey-Kramer post hoc test as appropriate. All analyses were performed using GraphPad Prism version 10 (GraphPad Software Inc., San Diego, CA, USA). Values were considered statistically significant at *P* < 0.05.

## Supplementary information

Supplementary information

Uncropped original western blots

## Data Availability

The data generated in this study and additional supporting data are available from the corresponding authors upon request.
