# Supplementary material for: IL-33-primed NLRP3 inflammasome in basophils drives IL-1β production and initiates atopic dermatitis inflammation
Source: Cell Death Discov. 2025 Jul 27;11:346. doi: 10.1038/s41420-025-02630-6 (PMC12297455; doi:10.1038/s41420-025-02630-6)

Supplementary figures

IL-33-primed NLRP3 inflammasome in basophils drives IL-1β production and initiates atopic dermatitis inflammation (Gunji et al.)

Supplementary Figure 1. Top 20 human cell types expressing IL1RL1 (ST2).

The top 20 human cell types ranked by *IL1RL1* expression levels, based on publicly available scRNA-seq data (Tabula Sapiens Consortium. Science 2022), are shown.

Supplementary Figure 2. Basophils express Il3ra and Fcer1a.

(A) An analysis of publicly available RNA-seq data (GSE116117) showing mRNA expression in mouse bone marrow and spleen (n = 1 for basophils of spleen, n = 3 for eosinophils, and n = 2 for others. P <0.0001 by a one-way ANOVA). (B) An analysis of publicly available RNA-seq data showing mRNA expression in human peripheral blood (n = 2 for $CD4^+$ terminal effector T cells, and n = 4 for others. P <0.0001 by a one-way ANOVA).

Supplementary Figure 3. Basophils express NLRP3 inflammasome components.

(A) An analysis of publicly available RNA-seq data (GSE116117) showing mRNA expression in mouse bone marrow and spleen (n = 1 for basophils of spleen, n = 3 for eosinophils, and n = 2 for others. P <0.0001 by a one-way ANOVA). (B) An analysis of publicly available RNA-seq data showing mRNA expression in human peripheral blood (n = 2 for $CD4^+$ terminal effector T cells, and n = 4 for others. P <0.0001 by a one-way ANOVA).

Supplementary Figure 4. A reanalysis of scRNA-seq data of an OXA-induced AD mouse model with a heat map.

Publicly available scRNA-seq data (GSE149121) were reanalyzed. A heat map is shown. Representative genes of each cluster are shown.

Supplementary Figure 5. A reanalysis of scRNA-seq data of an OXA-induced AD mouse model

with a violin plots.

Publicly available scRNA-seq data (GSE149121) were reanalyzed. Violin plots showing the expression of *Fcer1a, Mcpt8, Mcpt4, Cma1*, *Tpsb2,* and *Il18* in control and OXA-treated skins are shown.

Gunji, et al. Supplementary Fig. 1

Top 20 human cell types expressing *IL1RL1* (ST2)
(Tabula Sapiens Consortium, Science 2022)

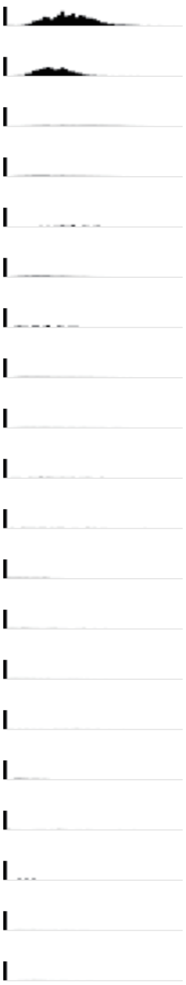

Cell type	Expression level	Cell number
Mast cell		6222
Basophil		1669
Capillary endothelial cell		15298
Cardiac endothelial cell		10092
Interstitial cell of Cajal		71
Luminal cell of prostate epithelium		10652
Endothelial cell of venule		52
Fibroblast of breast		5270
Endothelial cell of artery		3471
Bronchial smooth muscle cell		220
Colon endothelial cell		348
Basal cell of prostate epithelium		3495
Pancreatic stellate cell		438
Vein endothelial cell		4698
Endothelial cell of vascular tree		875
Progenitor cell of mammary luminal epithelium		1694
Myeloid dendritic cell		571
Pulmonary ionocyte		25
Endothelial cell of lymphatic vessel		2438
Vascular associated smooth muscle cell		5019

Gunji, et al. Supplementary Fig. 2

A Mouse bone marrow and spleen
(Choi, et al. Nucleic Acids Research 2018, GSE116177)

B Human peripheral blood
(Monaco, et al. Cell Reports 2019, GSE107011)

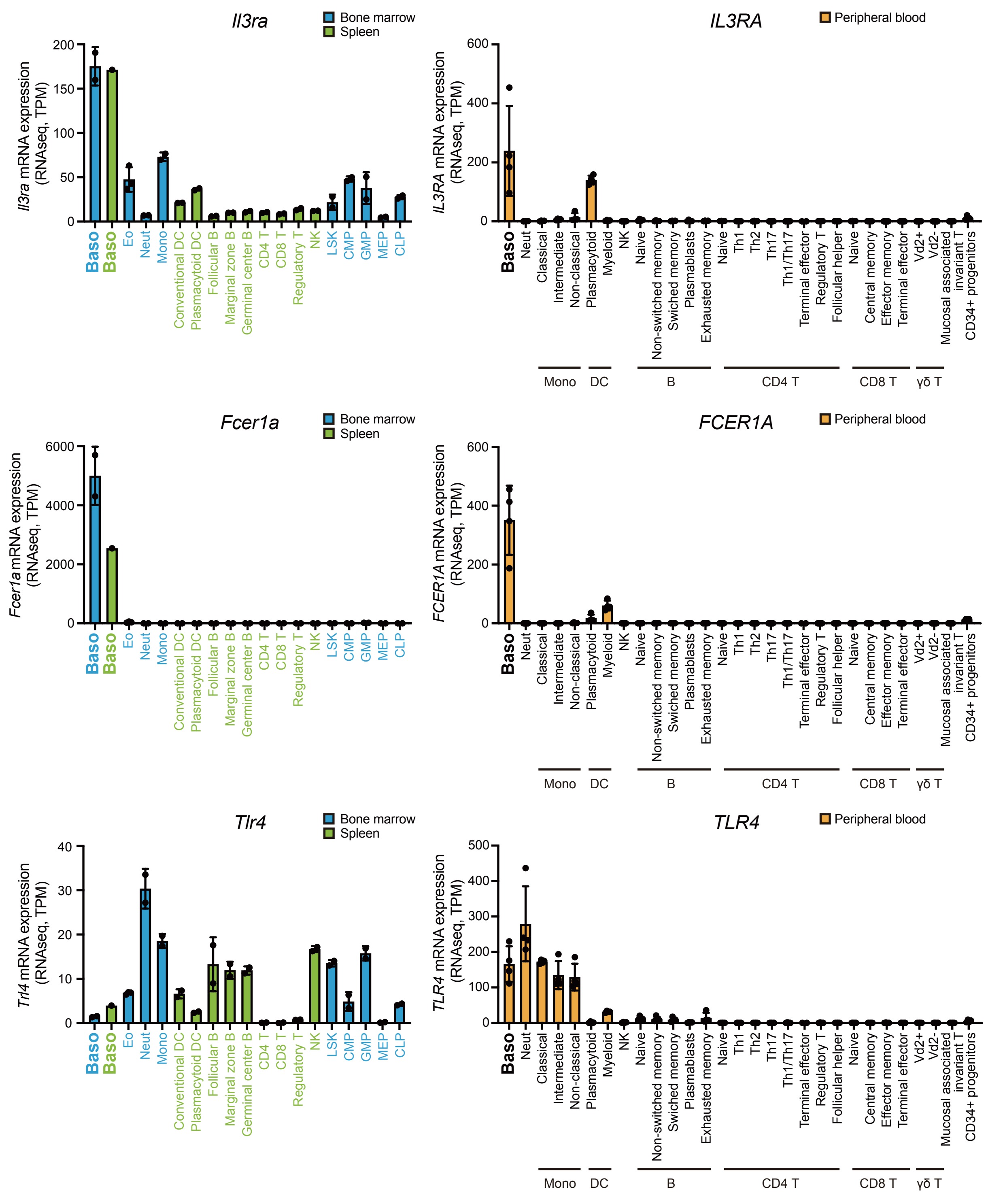

Gunji, et al. Supplementary Fig. 3

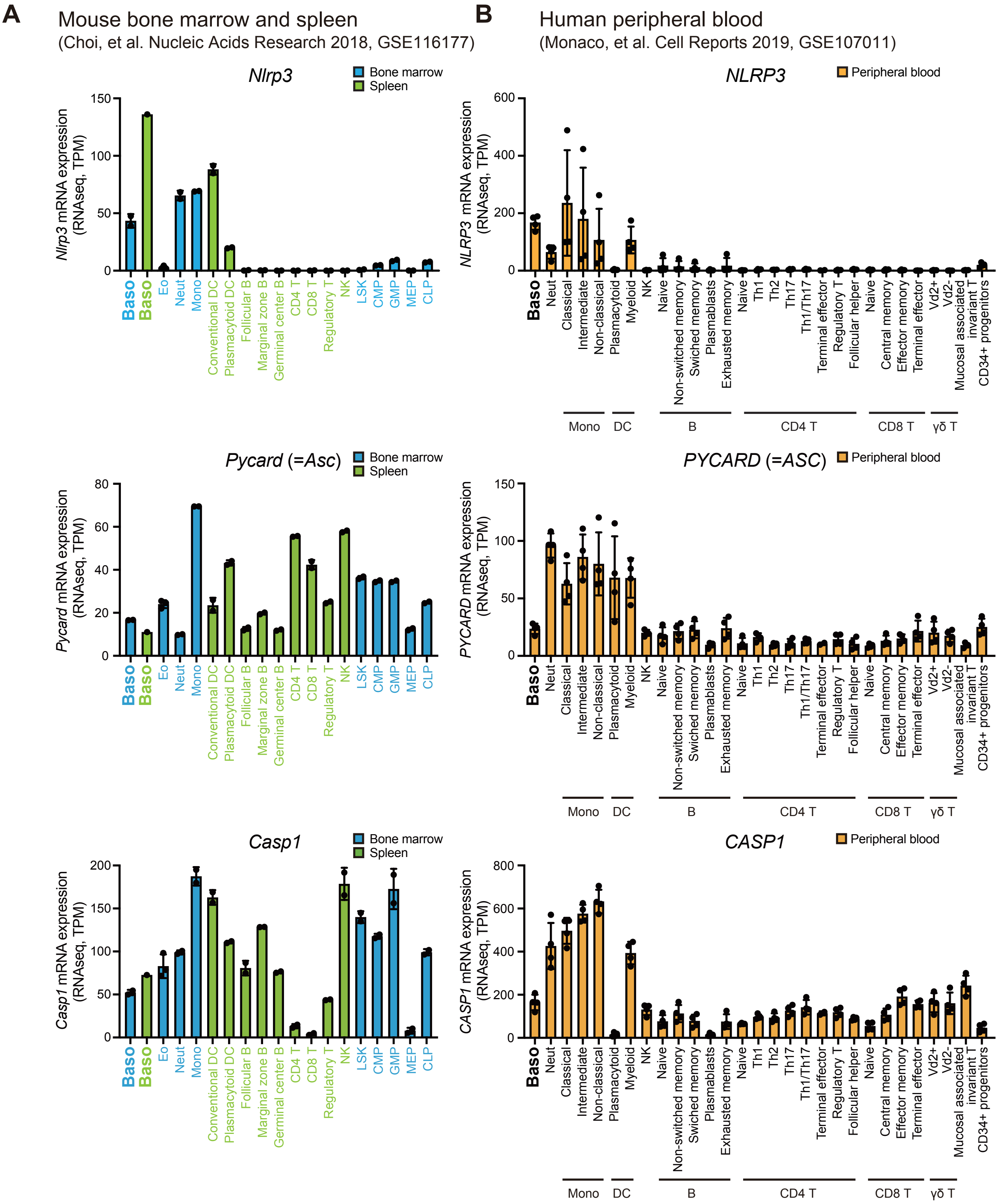

Gunji, et al. Supplementary Fig. 4

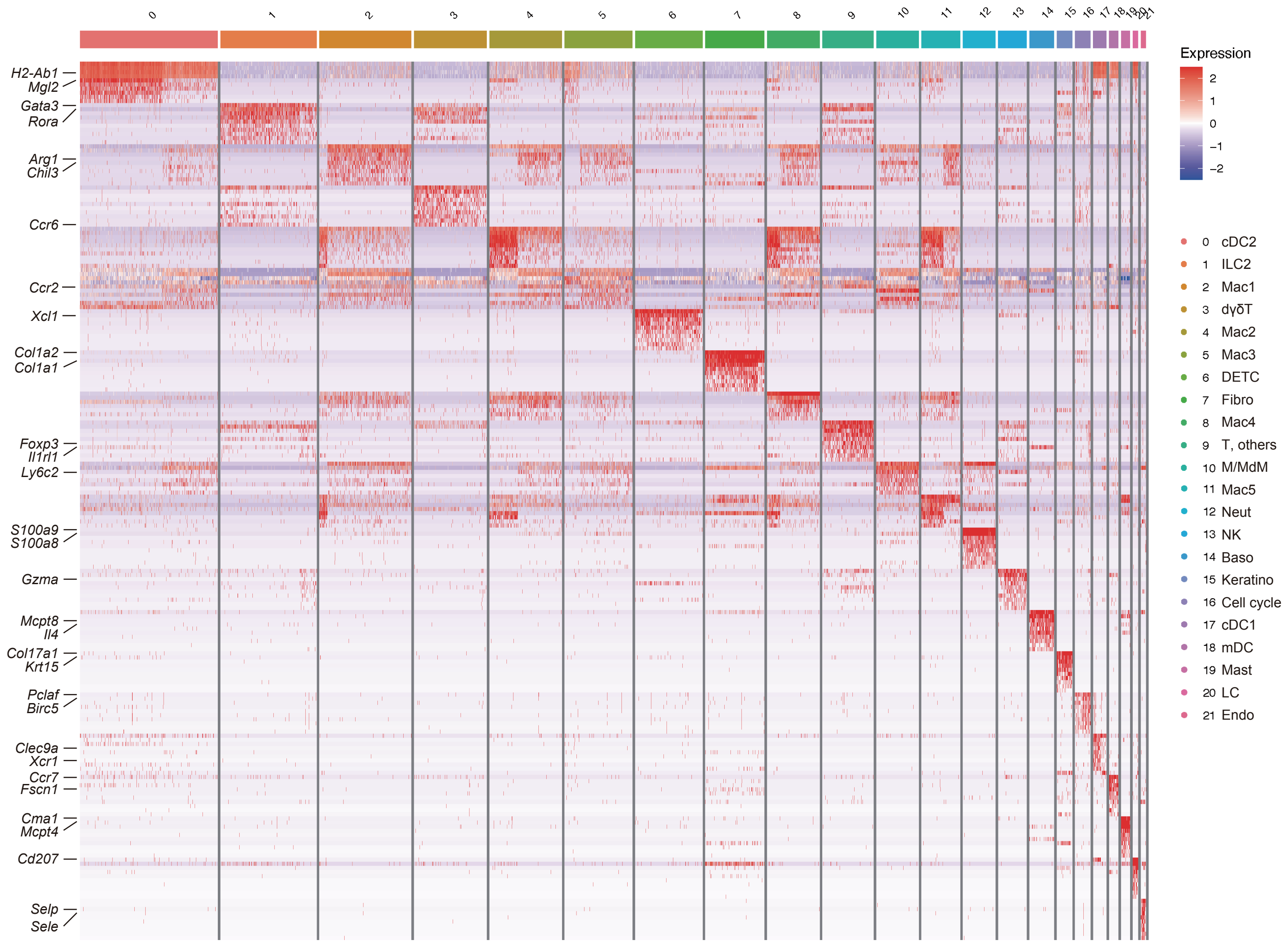

Gunji, et al. Supplementary Fig. 5

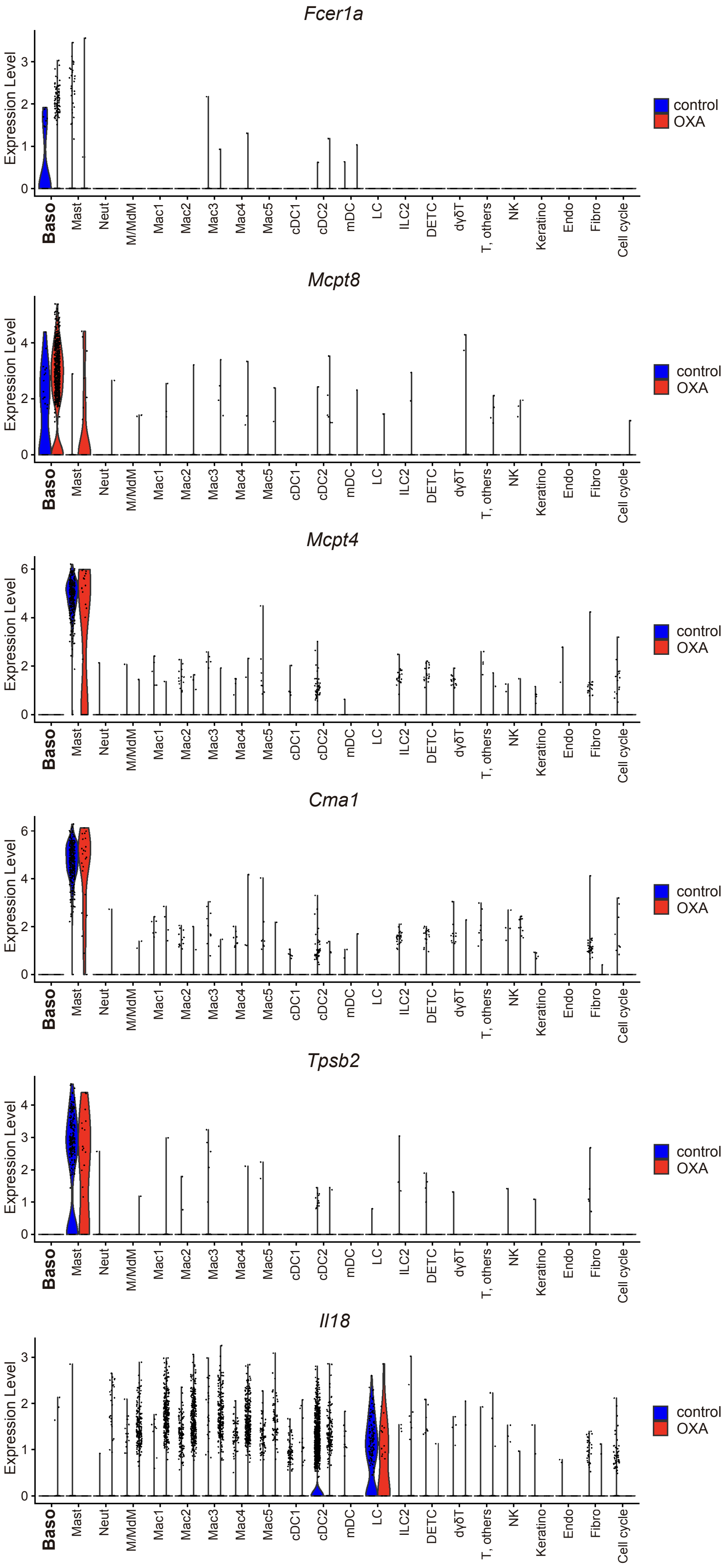

Supplement: Supplementary file 1 — Supplementary information [file 41420_2025_2630_MOESM1_ESM.pdf]
