# Supplementary material for: IL-33-primed NLRP3 inflammasome in basophils drives IL-1β production and initiates atopic dermatitis inflammation
Source: Cell Death Discov. 2025 Jul 27;11:346. doi: 10.1038/s41420-025-02630-6 (PMC12297455; doi:10.1038/s41420-025-02630-6)

Source Data
Uncropped Immunoblotting

Fig. 1I

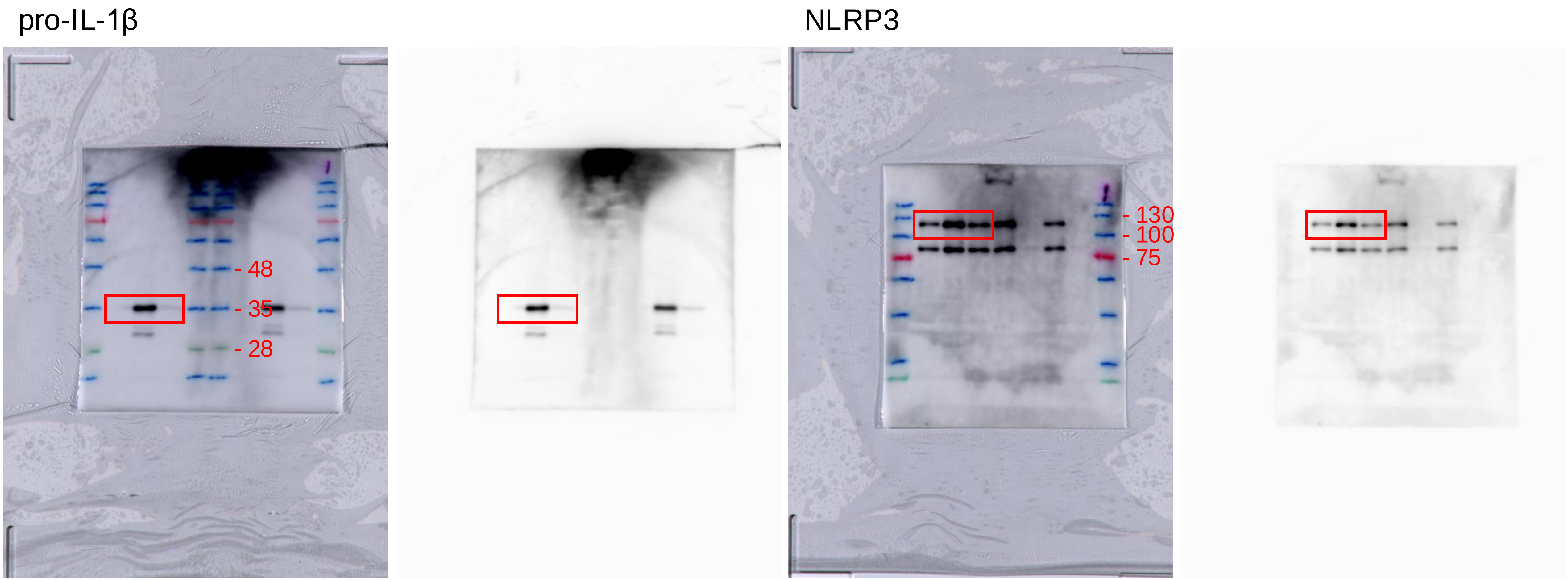

Fig. 1I

ASC

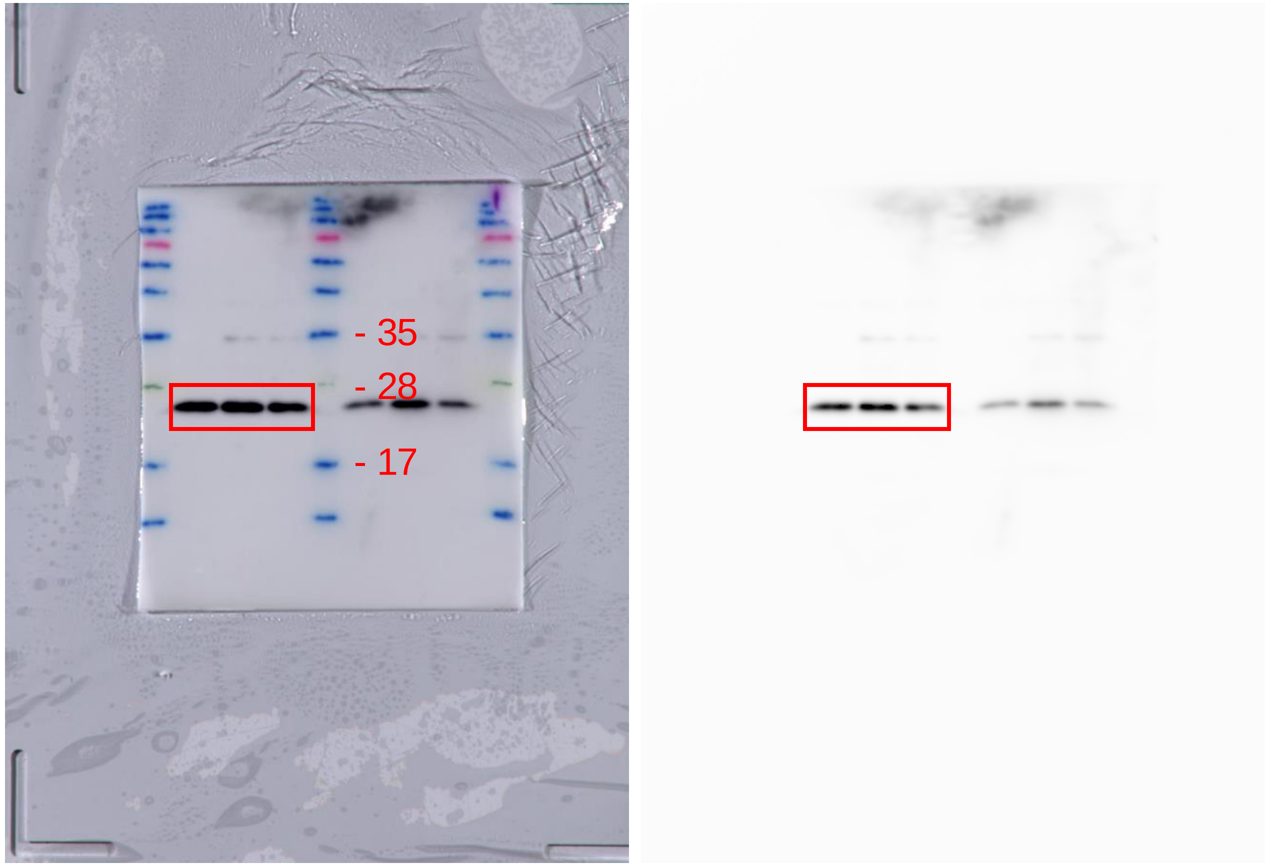

caspase-1

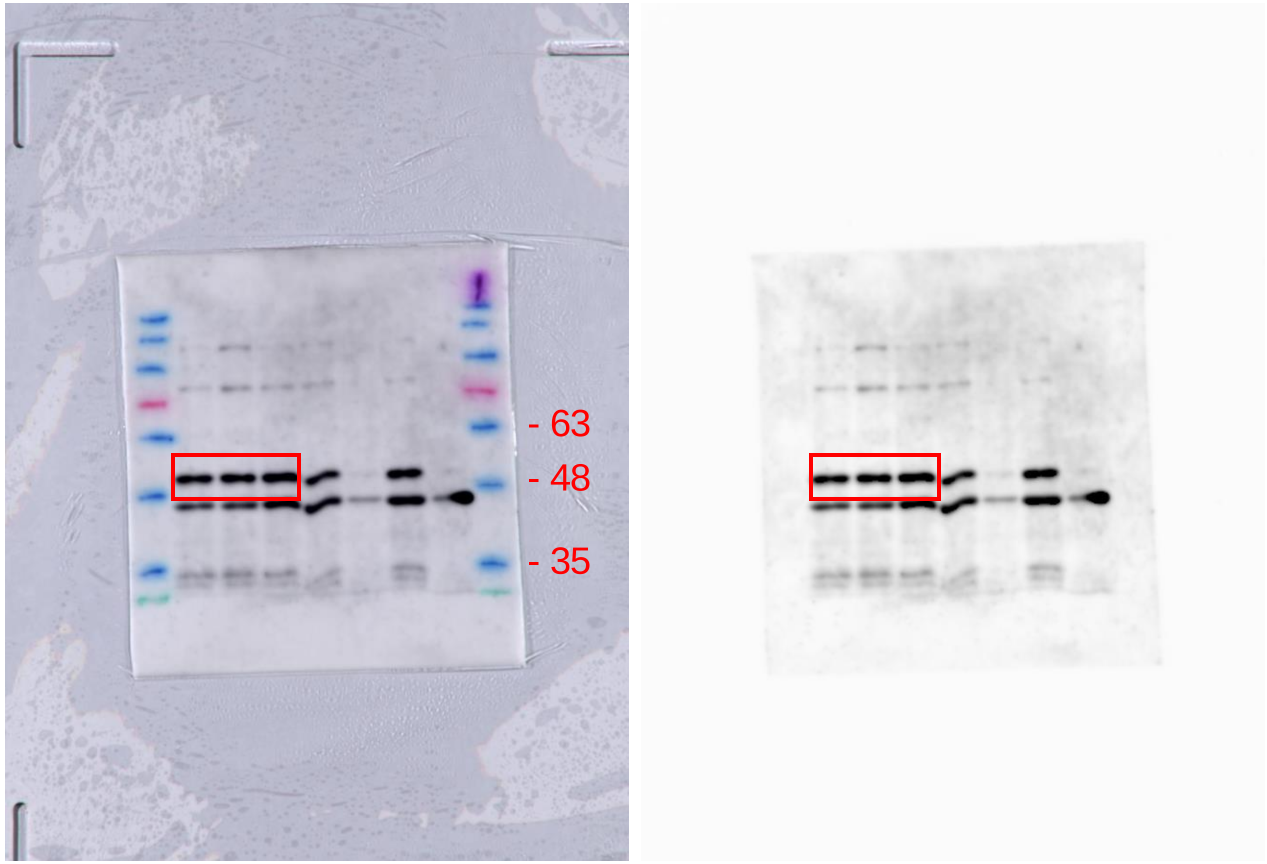

Fig. 1I

β-actin

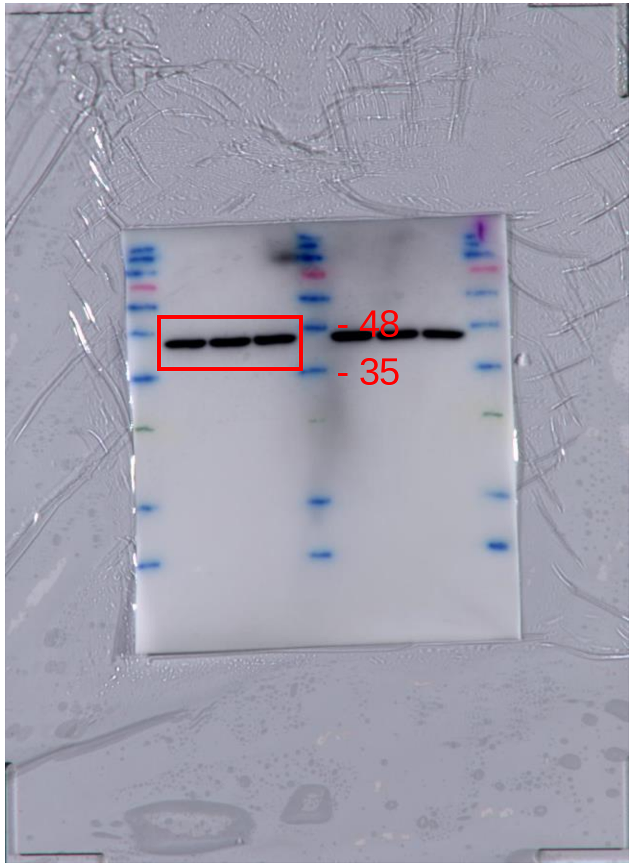

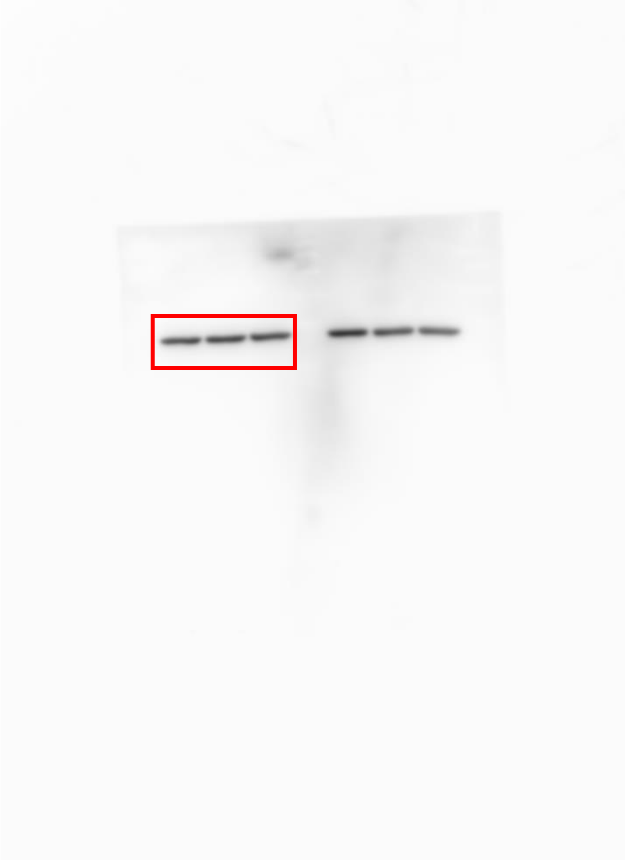

Fig. 3A

P-p65 NF-κB

p65 NF-κB

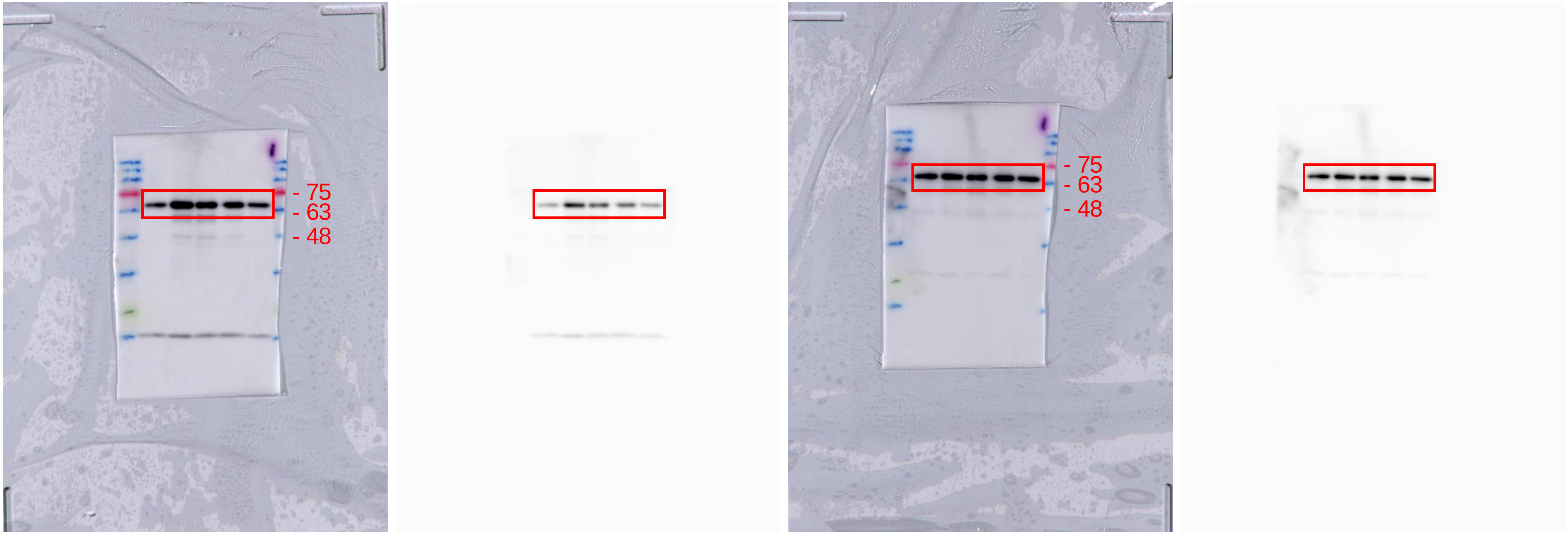

Fig. 3A

P-ERK

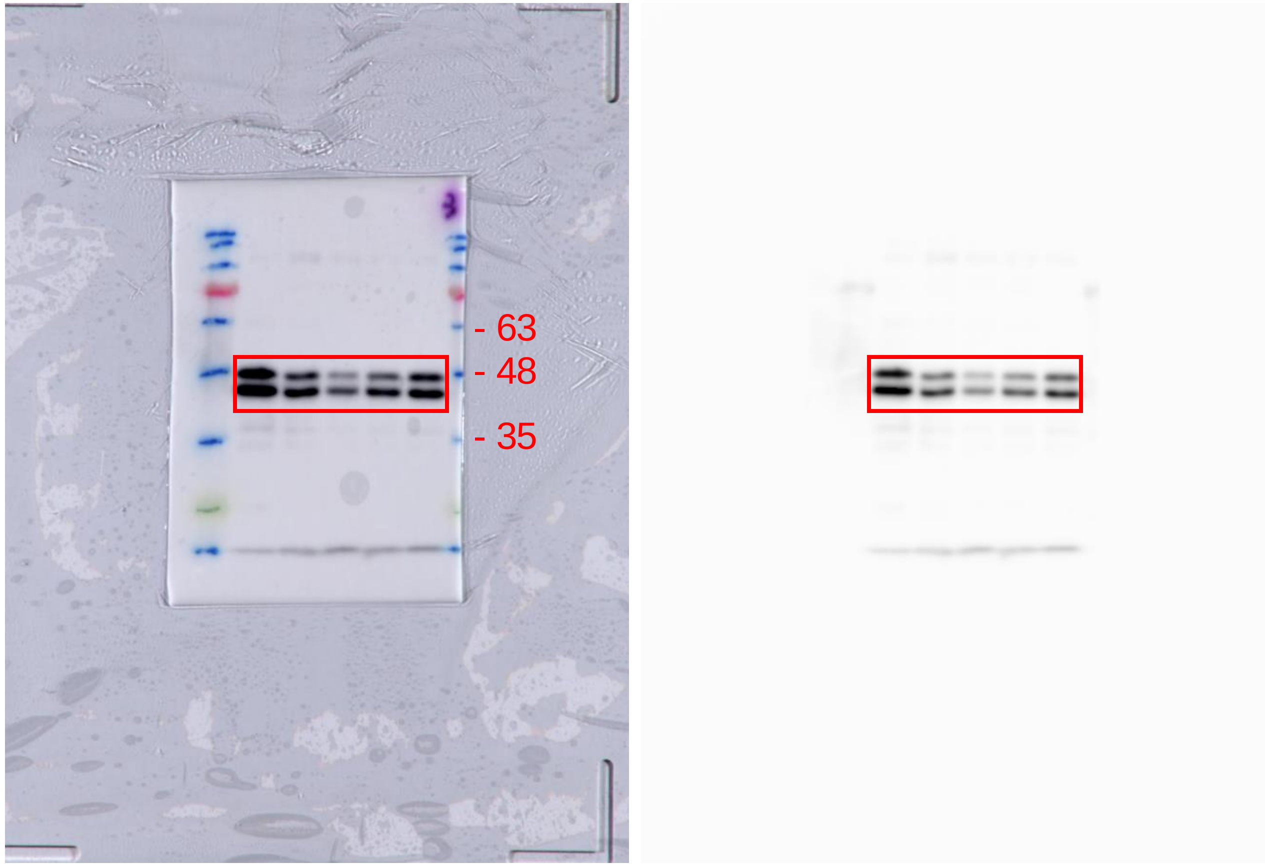

ERK

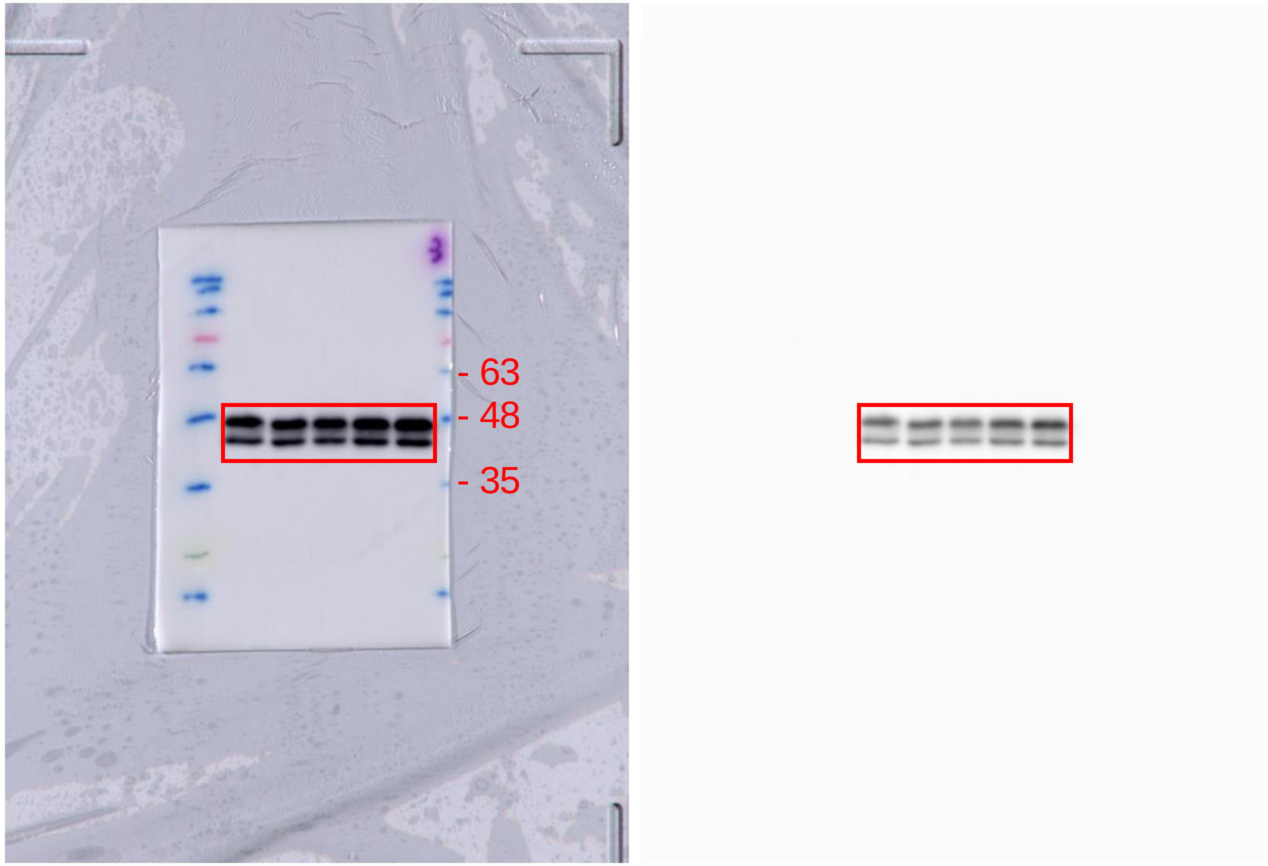

Fig. 3A

P-JNK JNK

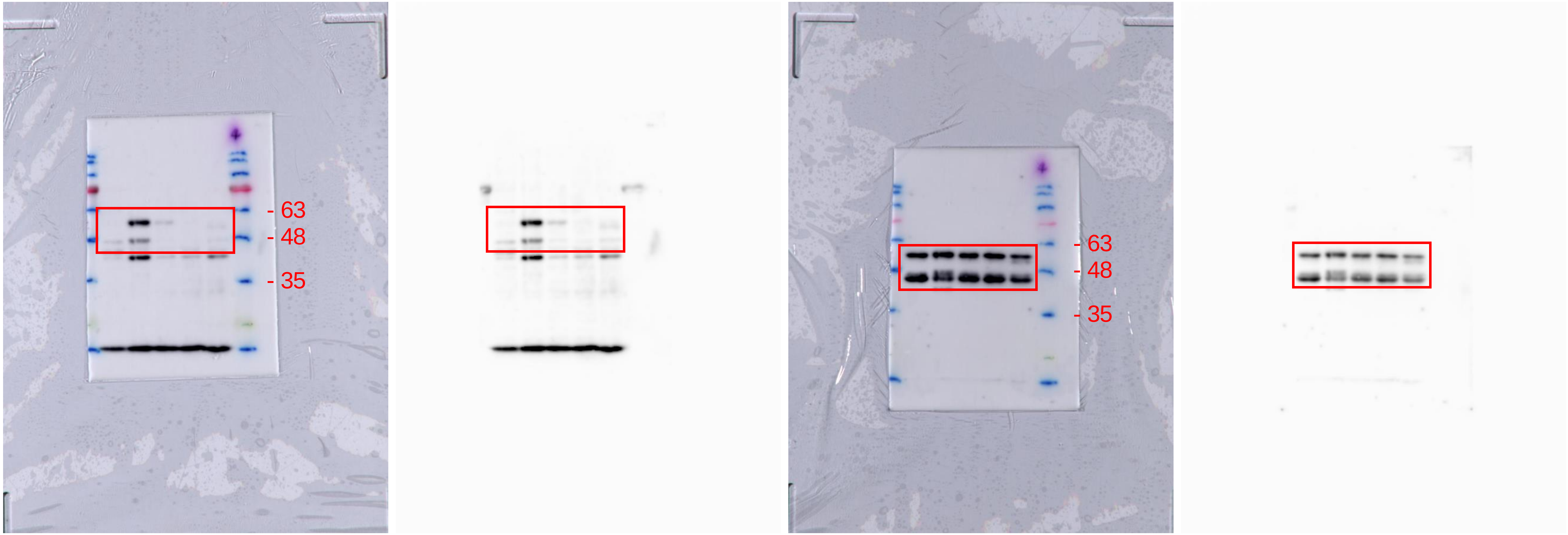

Fig. 3A

P-p38 MAPK

p38 MAPK

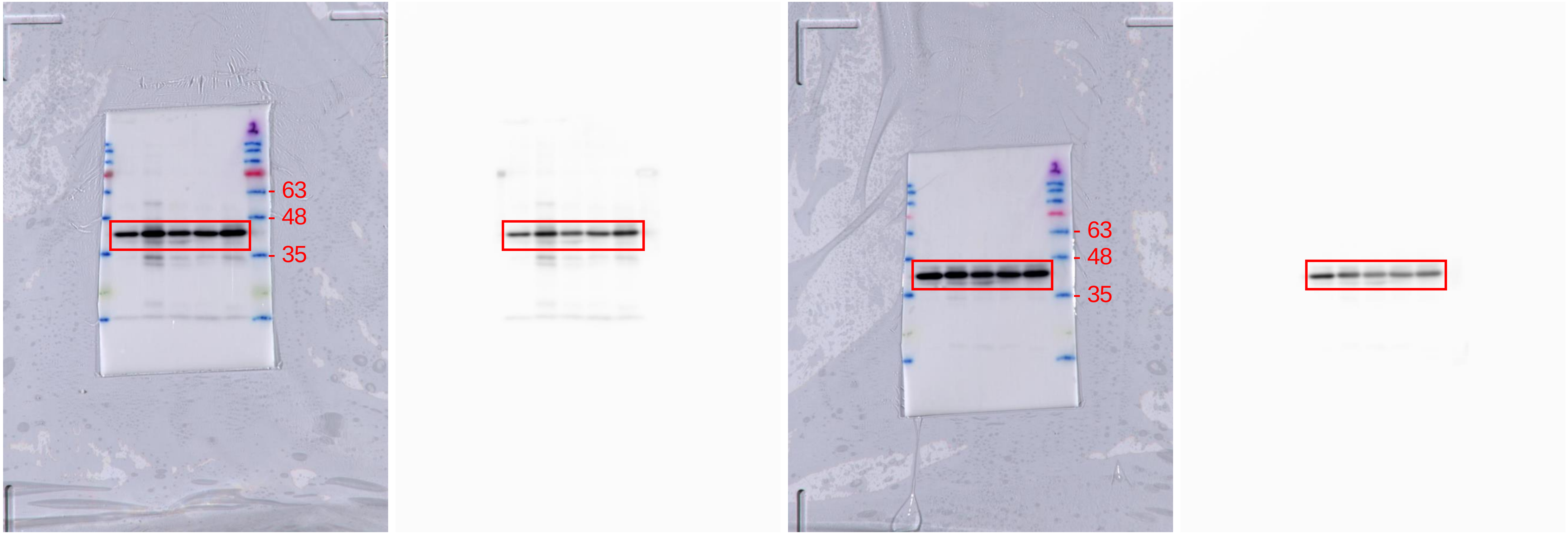

Fig. 3A

β-actin

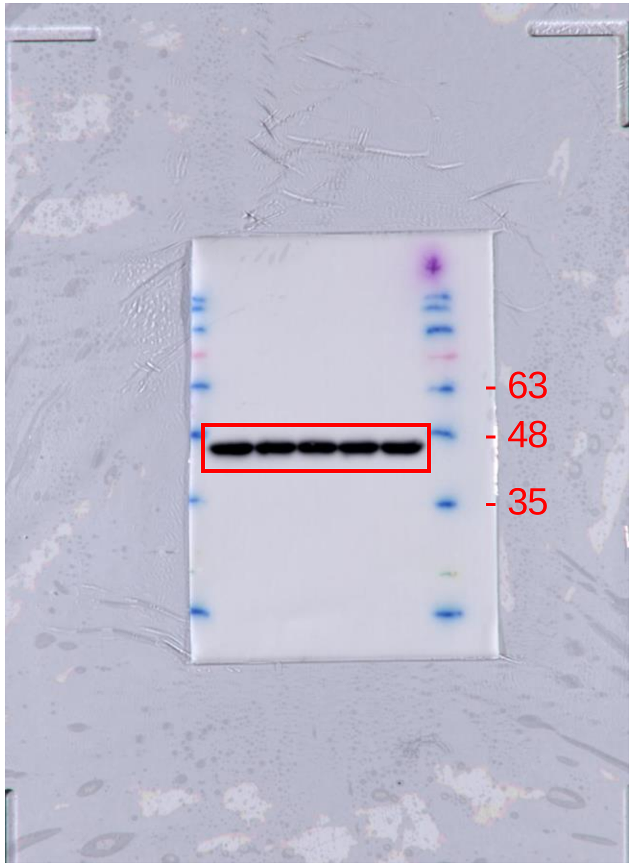

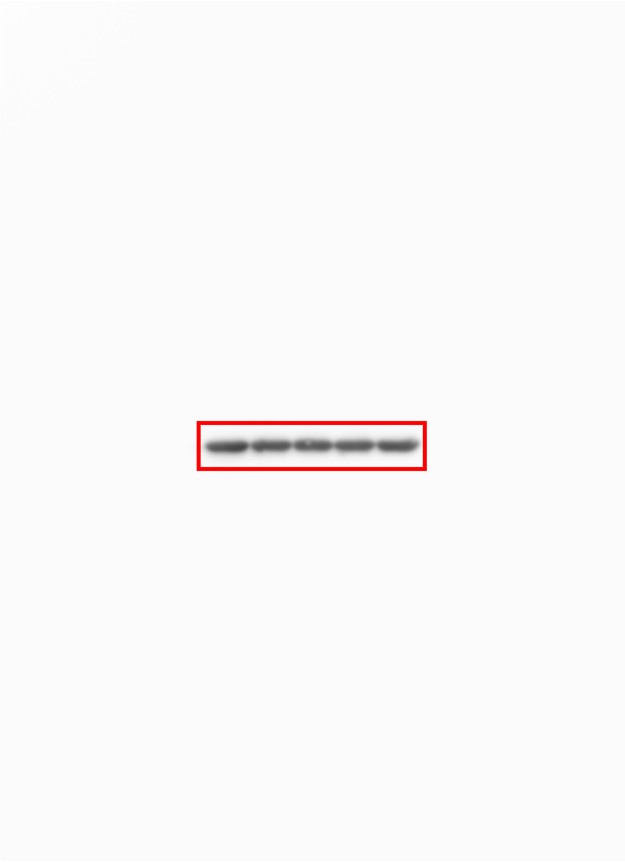

Fig. 4A

pro-IL-1β

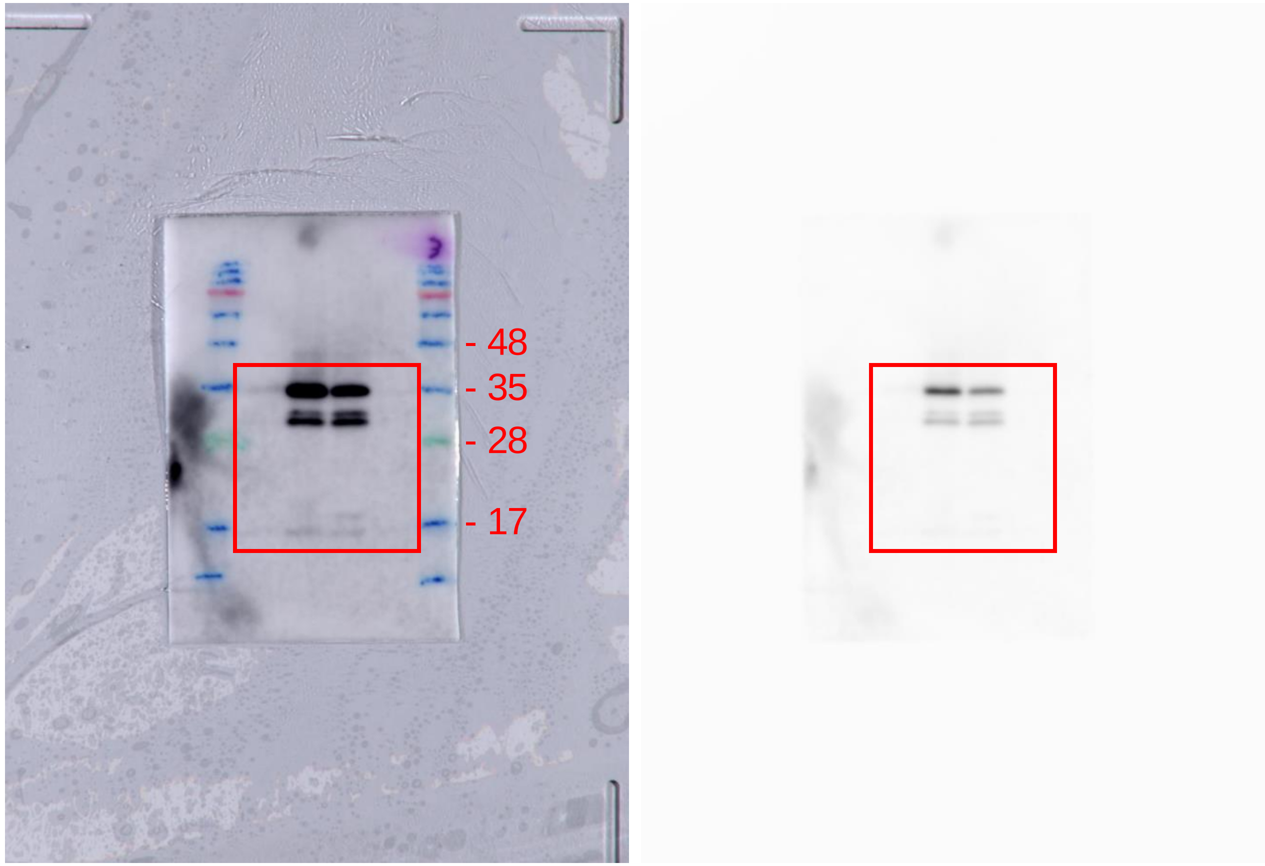

mature IL-1β

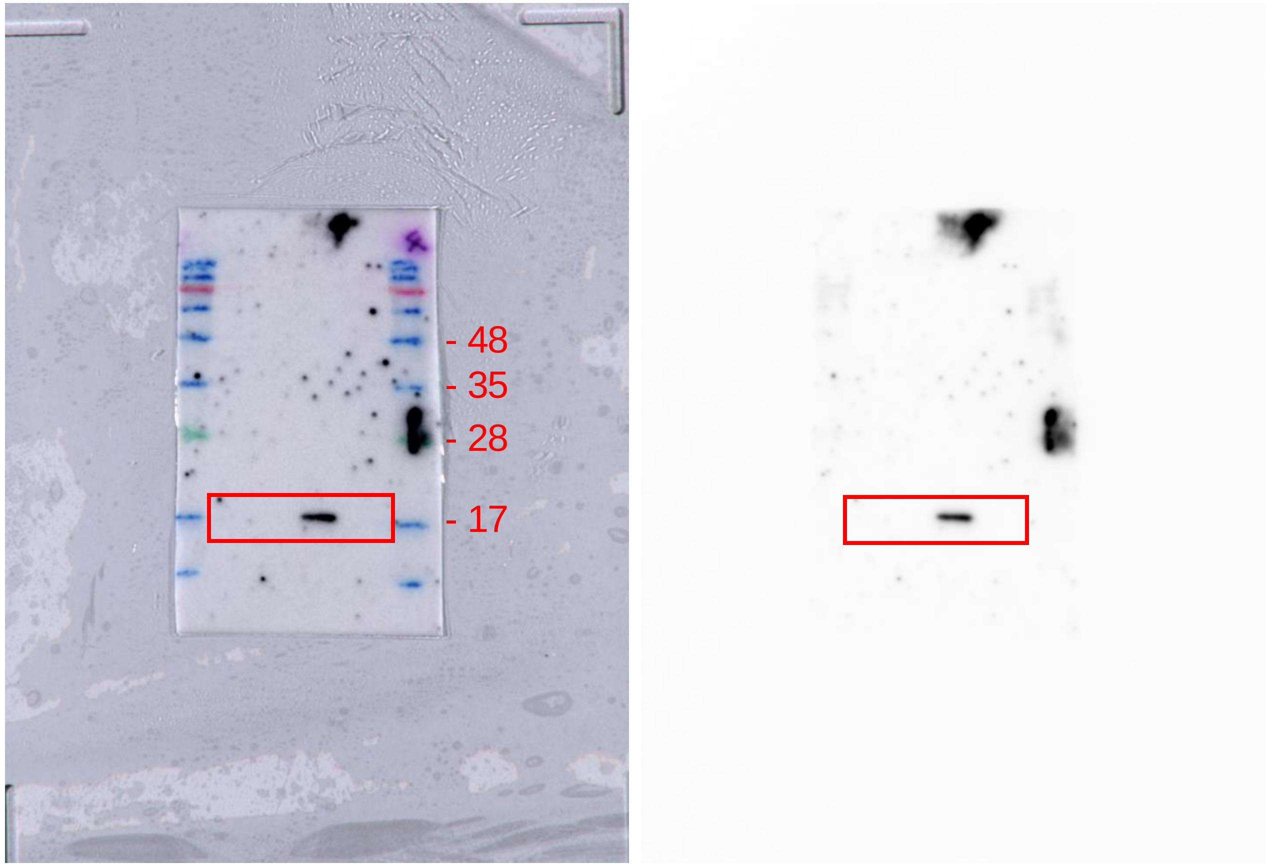

Fig. 4A

β-actin

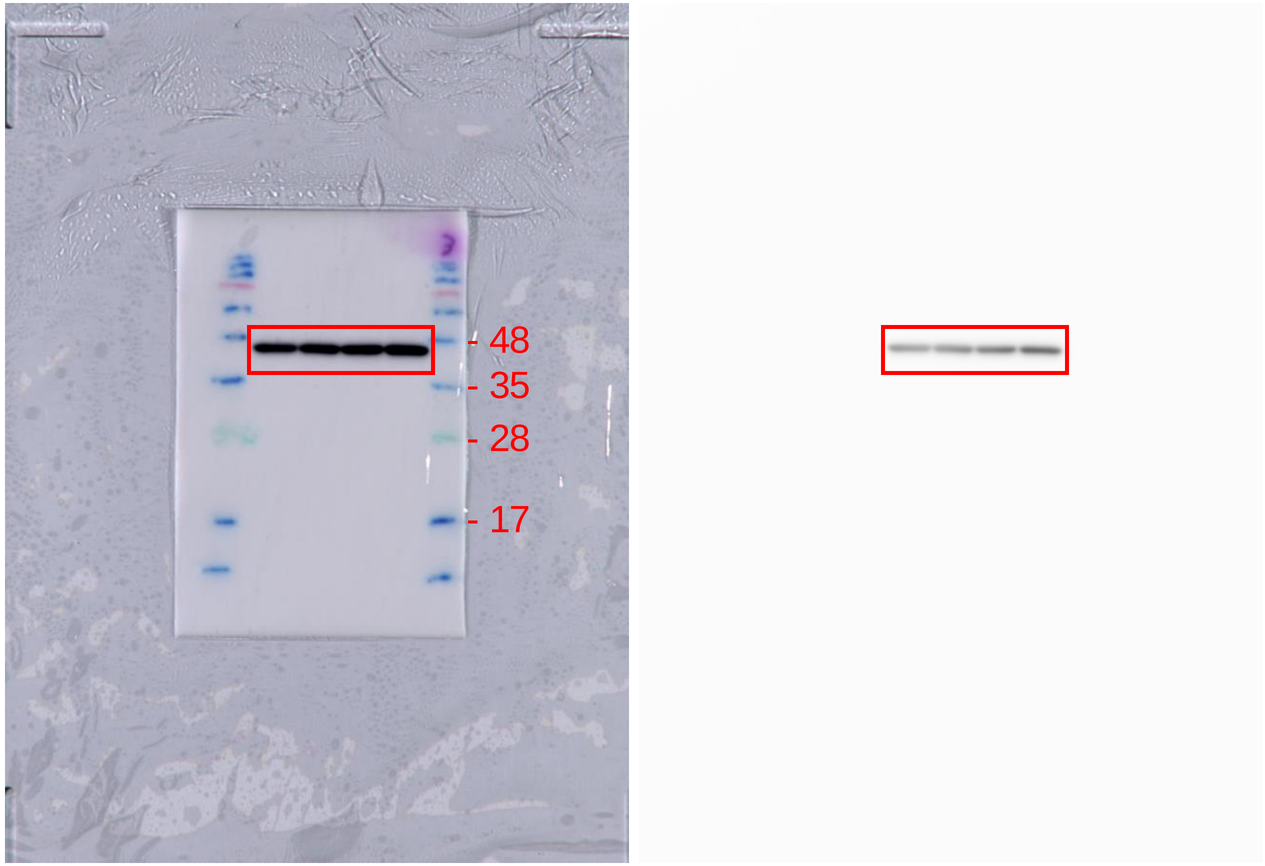

Supplement: Supplementary file 2 — Uncropped original western blots [file 41420_2025_2630_MOESM2_ESM.pdf]
